# Ramanujan expander networks for authenticated grayscale image encryption

**DOI:** 10.1371/journal.pone.0360934

**Published:** 2026-10-26

**Authors:** Asima Razzaque, Abdul Razaq, Dilshad Alghazzawi, Muhammad Ramzan, Hamiden Abd El-Wahed Khalifa

**Affiliations:** 1 Department of Mathematics and Statistics, College of Science, King Faisal University, Al-Ahsa, Saudi Arabia; 2 Department of Mathematics, Division of Science and Technology, University of Education, Lahore, Pakistan; 3 Department of Mathematics, College of Science and Arts, King Abdulaziz University, Rabigh, Saudi Arabia; 4 Department of Mathematics, Division of Science and Technology, University of Education, Lahore, Pakistan; 5 Department of Operations and Management Research, Faculty of Graduate Studies for Statistical Research, Cairo University, Giza, Egypt; Dalian Polytechnic University, CHINA

## Abstract

Image ciphers are usually proposed with a fixed battery of statistical indicators, and the numbers reported for competing designs are almost never produced under the same conditions, on the same pictures, with the same code. We address that gap for one family of designs and propose a cipher whose components come from provable combinatorics and standardised cryptography rather than a chaotic map. The scheme, RAPID-Gray, builds three keyed switching networks on the Lubotzky–Phillips–Sarnak Ramanujan graph X(29,41), restricts them to the image domain by cycle walking, interleaves them with two Cauchy maximum-distance-separable layers over $GF({\text{2}}^{\text{8}})$, applies a two-pass nonlinear feedback diffusion, and closes with AES-CTR whitening and an HMAC-SHA-256 tag over a fresh 96-bit nonce. Five recent designs were re-implemented without algorithmic alteration and evaluated with identical code on three medical and three general 256 × 256 grayscale images. All six recovered every plaintext exactly. Beyond that, the spread is wide: cipher entropy ranges from 6.9320 to 7.9976 bits, one collapses to an NPCR of 0.0015%, two produce unchanged ciphertexts under the tested one-bit key perturbation, and only the proposed scheme rejects tampered ciphertexts. Agreement on entropy and correlation says little; plaintext sensitivity, key sensitivity and integrity are where these designs actually separate. Three of the five reference designs left a step of their published description underdetermined; in each case a fixed, reversible resolution was adopted and is documented, so the comparison is between faithful re-implementations rather than between the published texts themselves.

## Introduction

### Motivation and problem statement

Digital images are bulky, highly redundant and strongly correlated in both spatial directions, and these properties have long been used to argue that they deserve dedicated encryption algorithms rather than a straightforward application of a standard block cipher. Whatever the merits of that argument, the practical stakes are not in doubt. Medical studies, biometric records and surveillance frames are transmitted and archived at scale, and for medical imaging in particular the relevant threat is not only disclosure but silent modification: a receiver that accepts an altered radiograph without noticing has suffered a failure that no measure of ciphertext randomness prevents. An image cipher intended for such data must therefore be judged on three properties at once — confidentiality, sensitivity of the ciphertext to both the plaintext and the key, and the ability to detect tampering — and the third of these is almost universally absent from the literature the present work engages with.

The evaluation problem is just as pressing as the design problem. Comparison tables in image-encryption papers are typically assembled from numbers reported elsewhere, computed on other images, at other resolutions, with other implementations of the same statistic. A local entropy computed on 16 × 16 blocks and one computed on 44 × 44 blocks are not the same quantity, and an NPCR averaged over many random single-bit flips is not the same as one obtained from a single flip in the least significant bit of the first pixel. A scheme is almost never re-implemented by anyone other than its authors, so the difference between what an algorithm does and what its description says it does is rarely visible. The question this paper asks is what happens when that difference is made visible: when six schemes are implemented in one program, run on the same six images, and measured with one copy of each test routine.

To state the motivation explicitly, this work addresses three specific gaps rather than a general wish for another cipher. The first is a security gap. The designs engaged with here protect confidentiality only, so a receiver handed a modified radiograph decrypts it into a plausible but wrong picture and has no mechanism for detecting that anything was changed; for medical imaging this is a clinically relevant failure mode, and no amount of ciphertext randomness repairs it. The second is a foundational gap. The mixing behaviour of a chaos-based confusion stage is supported by simulation evidence — bifurcation diagrams, numerically estimated Lyapunov exponents — and is modified in practice by finite-precision arithmetic, whereas the mixing rate of a walk on an expander graph, the avalanche of a maximum-distance-separable matrix and the security of AES and HMAC are quantities backed by theorems or standards. The third is a methodological gap. Comparison tables are assembled from numbers computed by different authors, on different images, at different resolutions and with different definitions of nominally the same statistic, so they cannot support the ranking claims that are routinely built on them. The paper therefore asks two questions: can a competitive image cipher be assembled entirely from components carrying provable or standardised guarantees, and what happens to the published picture of this field when several recent schemes and one new one are re-implemented in a single program and measured with a single copy of every test? The design contribution answers the first question and the controlled comparison answers the second.

### Related work

The modern image-encryption literature descends from two early proposals: Matthews’ chaotic stream cipher [1] and Fridrich’s permutation–diffusion architecture built on two-dimensional chaotic maps [2]. Fridrich’s template — a confusion stage that rearranges pixel positions using the orbit of a chaotic map, followed by a diffusion stage that spreads a change in one pixel across the frame — has organised several thousand subsequent designs. The template survives because it is convenient: chaotic recurrences are cheap to iterate, their outputs look random to the eye, and their sensitivity to initial conditions is easy to present as a cryptographic virtue. Variations have multiplied along every axis of the template: three-dimensional and higher-dimensional chaotic maps [3,4], cosine-transform and sine-logistic compositions that enlarge the parameter ranges over which the dynamics remain chaotic [5], bit-plane decompositions that move the permutation from the pixel level to the bit level, and S-boxes generated from chaotic orbits, algebraic structures or, as in one of the schemes compared here, fuzzy set theory, to supply the nonlinearity. General surveys of the resulting design space are available [6,7].

The critical literature has grown alongside the constructive one. Alvarez and Li set out, two decades ago, a list of basic requirements [8] — a defined key space, key sensitivity, resistance to chosen-plaintext attack — that most chaos-based proposals of the time did not meet, and periodic reviews since then have found the situation improving only slowly [9]. Preishuber and co-workers showed that a large collection of published schemes pass the standard statistical battery while failing elementary security requirements, and that the battery itself is largely insensitive to the differences that matter [10]. A steady stream of cryptanalyses has confirmed the diagnosis in individual cases: schemes whose reported entropy, correlation and NPCR are indistinguishable from those of AES have been broken with a handful of chosen plaintexts — Solak and co-workers’ recovery of Fridrich’s own scheme is an early and representative example [11] — usually because the keystream was independent of the plaintext in a way the designer did not intend, or because a permutation and a substitution presented as independent layers compose into a single equivalent map. Li and colleagues’ year-in-review surveys of broken ciphers, and Muhammad and Özkaynak’s review of the design pathology behind them, document the pattern at scale [12,13].

Three ingredients of the present work come from outside that literature. Expander graphs — sparse graphs on which random walks mix rapidly — are a mature subject in combinatorics [14,15], with the Ramanujan graphs of Lubotzky, Phillips and Sarnak, and independently of Margulis, achieving the optimal spectral gap permitted by the Alon–Boppana bound [16,17]; their cryptographic use to date has centred on hash functions built from expander walks [18] rather than on permutation generation for image encryption. Maximum-distance-separable matrices are the standard diffusion tool of block-cipher design [19], where the branch number of the AES MixColumns matrix quantifies exactly the avalanche guarantee that image ciphers claim informally [20]; Cauchy constructions give MDS matrices of any size over any field [21]. And the composition of encryption with message authentication is settled theory: encrypt-then-MAC yields authenticated encryption from any secure cipher and any secure MAC [22,23], a result that the image-encryption literature has, with few exceptions, not absorbed. The present design imports all three.

The field has continued to develop since these strands were established, and three recent directions bear directly on the present work. First, the chaotic source itself has moved towards memristive and higher-dimensional systems with richer dynamics, for instance the memristor-based four-dimensional chaotic system of Zhang and co-workers [24], and the discrete Izhikevich star-coupled neuron network of Zhang and colleagues [25], realised on FPGA hardware; such systems enlarge the parameter space of the keystream generator, but they do not by themselves address the finite-precision and key-sensitivity questions examined in the Discussion. Second, and closer to the argument made here, several 2024–2026 designs have begun to combine encryption with authentication rather than treating confidentiality as the whole problem: the pixel-adaptive diffusion scheme of Gong and co-workers [26] pairs encryption with a mechanism for establishing the origin of a received image, the computational-ghost-imaging construction of Hu and co-workers [27] embeds an authentication image in the encrypted output, and the quantum-domain constructions of Zhou and co-workers [28] pursue a related goal in a different representation. These works confirm that the integrity gap identified here is recognised in parts of the field; the mechanisms adopted are image-domain or optical-domain constructions rather than a standard message authentication code with a composition theorem behind it, and none of them is among the five schemes re-implemented here. Third, the adjacent problem of protecting ownership rather than content has produced attack-resilient watermarking frameworks [29], which address a different threat model but share the underlying observation that a receiver needs verifiable evidence about an image and not merely a scrambled version of it.

A parallel line of work develops the chaotic sources themselves within memristive neural architectures. Fractional-order dual-memristor Hopfield networks with hidden multi-scroll attractors have been realised on FPGA hardware and applied to image encryption [30], discrete memristive neuron maps exhibiting extreme multistability have been proposed for dual-field applications [31], and the same family of networks has been turned to time-series forecasting [32]. We cite these as evidence of how rapidly the dynamical repertoire available to a designer is expanding. The argument of the present paper is orthogonal to that expansion rather than opposed to it: a richer attractor structure enlarges the space of keystreams a designer can draw on, but the properties that separated the six schemes compared here — whether a one-bit key change propagates through a finite-precision implementation, and whether a receiver can detect a modified ciphertext — are not properties of the underlying dynamical system at all, and a more complex attractor does not by itself supply either of them.

The same literature has broadened beyond encryption into the modelling of neuronal and physiological processes and into hardware realisation. Memristor–neuron coupled systems have been used to model pain modulation [33], dual-memristor Sprott-C systems and hyperbolic-tangent-regulated memcapacitor networks have been analysed for multi-scroll generation and implemented on digital signal processors [34,35], locally active memristive Chialvo maps have been applied to the dynamic modelling of bipolar disorder [36], and capacitively coupled heterogeneous memristor systems have been shown to sustain coexisting chaotic and spiking regimes [37]. Closer to the security application, a lightweight surveillance-video protection scheme has been built on a modified DES structure driven by chaotic keys [38], which is of particular interest here because it combines a standard block-cipher structure with a chaotic key source rather than replacing the cipher altogether. That hybrid is closer in spirit to the present design than a purely chaotic construction, and the difference that remains is the one this paper is about: a keyed standard primitive supplies a security argument that a chaotically generated key schedule alone does not, and neither supplies integrity unless a message authentication code is added.

Two further recent directions bear on the present study. For medical Internet-of-Things applications, Jin and co-workers combine a dynamic Hill cipher, whose key matrix varies over time, with logistic-chaos pixel scrambling [39], and Yu and co-workers realise four-dimensional discrete memristive hyperchaotic maps on FPGA hardware and apply them to image encryption [40]. Beyond encryption itself, robust watermarking based on conditional diffusion models [41] and robust image hashing for copy detection [42] provide complementary mechanisms for ownership and image verification. These address threat models different from authenticated encryption, but they illustrate the same need to distinguish confidentiality from verification and integrity.

### The proposed scheme at a glance

RAPID-Gray is an authenticated grayscale image cipher whose overall structure is shown in Fig 1. A 256-bit master key and a fresh 96-bit nonce are expanded by HKDF-SHA-256 into four independent subkeys governing permutation, diffusion, whitening and authentication. Position confusion is performed by three keyed switching networks — 24, 23 and 24 switch layers respectively — built on the Lubotzky–Phillips–Sarnak Ramanujan graph X(29,41), whose 68 880 vertices are restricted to the 65 536 pixel positions by cycle walking so that the ciphertext is exactly as long as the plaintext. Between consecutive switching stages, an 8 × 8 Cauchy MDS matrix over $GF({\text{2}}^{\text{8}})$ with branch number nine mixes byte values, so that positional and value mixing alternate. A two-pass nonlinear feedback layer built on the AES S-box, run once forward and once backward, makes every output byte depend on every input byte. The transformed frame is whitened with AES-256 in counter mode under a nonce-derived initial value, and the ciphertext, nonce and header are authenticated with HMAC-SHA-256 in the encrypt-then-MAC arrangement. Decryption verifies the tag in constant time before performing any inverse transformation or releasing any plaintext, and rejects any tampered ciphertext or wrong key outright. No layer of the construction relies on a chaotic map: the design of the switching networks is motivated by the spectral gap of the underlying graph, and their distributional behaviour is assessed empirically; the avalanche of the MDS layers is guaranteed by their branch number; and the security of the outer layers rests on the standard assumptions on AES and HMAC.

**Fig 1:**
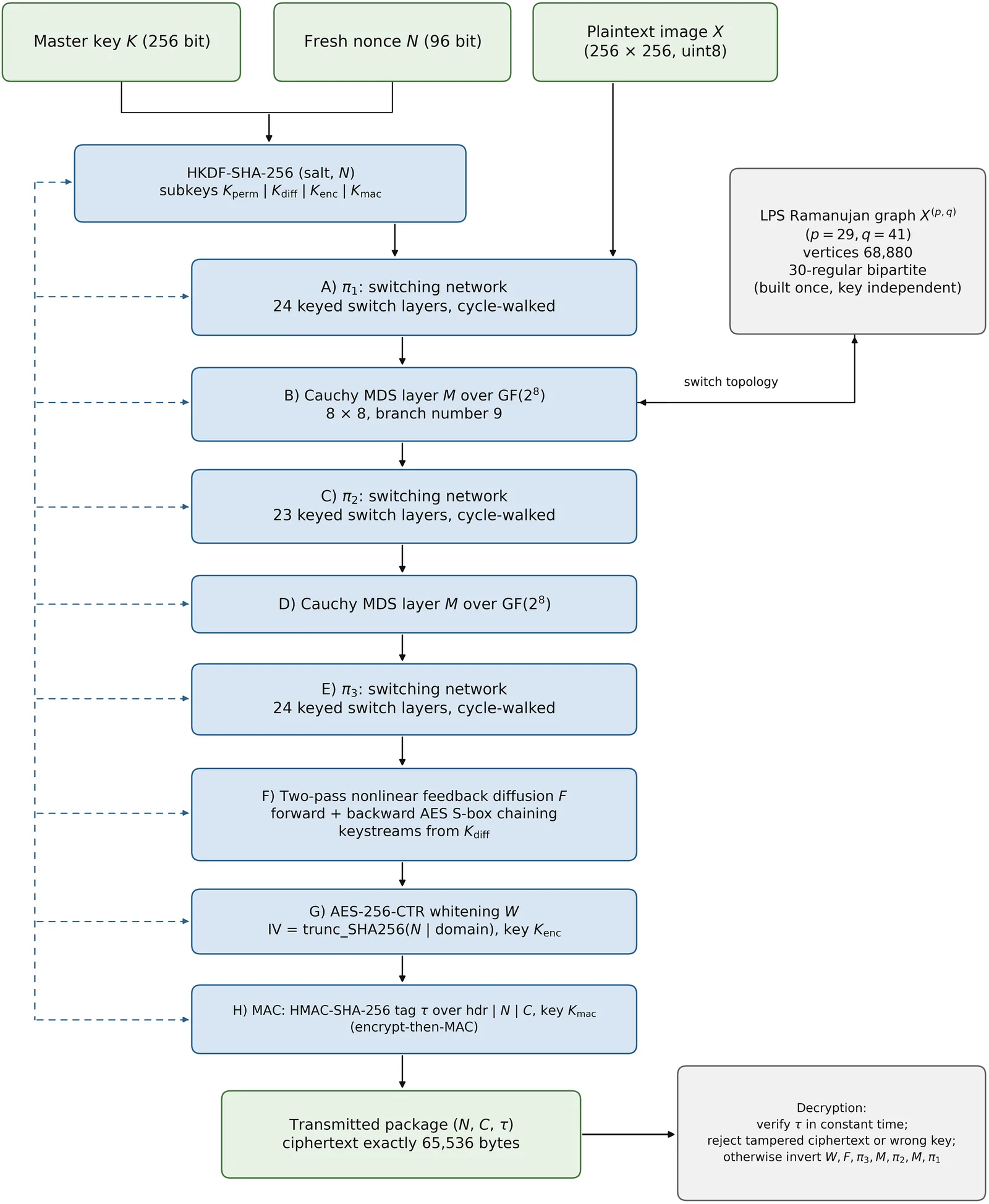
Overall structure of the proposed RAPID-Gray scheme. The master key and a fresh nonce are expanded by HKDF-SHA-256 into four subkeys. Three keyed switching networks on the LPS Ramanujan graph X(29,41) (cycle-walked to the 65 536 pixel positions) alternate with two Cauchy MDS layers over $GF({\text{2}}^{\text{8}})$; a two-pass nonlinear feedback diffusion, AES-256-CTR whitening and an HMAC-SHA-256 tag complete the pipeline. Decryption verifies the tag in constant time before performing any inverse transformation or releasing any plaintext, and rejects tampered ciphertexts and wrong keys. The figure is a structural overview only; the encryption and decryption procedures are specified step by step as Algorithms 1 and 2 in the Methods, with each step cross-referenced to the equation that defines it.

### Contributions

The contributions of the paper are the following. First, we give a complete specification of the proposed cipher, including the explicit construction of the underlying Ramanujan graph, the cycle-walking argument that restricts a permutation of 68 880 vertices to the 65 536 pixel positions without padding, and the reversibility of each layer; the design is, to our knowledge, the first image cipher to use keyed switching networks on an optimal expander as its confusion stage. Second, we report a controlled six-way comparison in which five recent designs — a fuzzy S-box scheme, a quantum-operator dynamic-matrix cipher, a multi-level bit-plane encryption core, an ISL-HMC dual-S-box scheme and a five-round permutation–diffusion–substitution structure — were re-implemented without algorithmic alteration and measured with a single copy of every statistic, covering histogram uniformity, global and local entropy, adjacent-pixel correlation, plaintext differential behaviour over one hundred independent single-bit trials per image, one-bit key sensitivity, a screening subset of NIST SP 800−22, and ciphertext integrity. Third, the comparison yields findings that no aggregation of published numbers could have produced: two of the five reference schemes produce unchanged ciphertexts under a one-bit key change, a third does so on exactly half of the test images, one has essentially no plaintext sensitivity, and none can detect a modified ciphertext. Fourth, we report the trial-level behaviour of every scheme, not only the trial means, and in doing so record the one measure on which the proposed scheme is not the best of the six: the spread of its differential response across individual trials.

The Results section presents the measurements; the Discussion interprets them; a separate section states the limitations; the Methods section gives the full construction of the proposed scheme, the resolution notes for each reference implementation, and the definitions of every statistic used.

## Results

### Test material and experimental protocol

Six standard grayscale images were used throughout: three medical studies (a knee MRI slice, an ankle CT slice and a mid-volume head MRI slice) and three general photographic or textural images (Cameraman, Coins and Rice). Every image was converted to 8-bit grayscale and resampled to exactly 256 × 256 pixels, so that all schemes operate on byte strings of identical length and the statistics are directly comparable. Table 1 summarises the six schemes and Table 2 gives the baseline statistics of the plaintexts. The three medical images are the most redundant of the set; the head MRI slice, which is largely black background, has a global entropy of only 3.6448 bits, and the ankle CT slice is barely higher at 3.6465. Cameraman and Rice sit close to 7 bits. All six plaintexts show adjacent-pixel correlations above 0.87 in every direction, and above 0.98 in two cases, which is the redundancy that any image cipher has to destroy.

**Table 1:** The six schemes compared in this study. Only the proposed scheme uses a nonce and only the proposed scheme authenticates its output.

Label	Scheme	Principal mechanism	Key material	Auth.
Proposed	RAPID-Gray	Ramanujan expander switching networks, two Cauchy MDS layers, two-pass nonlinear feedback diffusion, AES-CTR whitening	256-bit master key + 96-bit nonce	Yes
S2	Fuzzy S-box scheme [43]	Fuzzy-derived 16 × 16 S-box, bit-plane circular shifts and pairwise plane mixing, SHA-512 of the plaintext, chained XOR diffusion	Plaintext digest (key optional)	No
S3	Quantum-operator scheme [44]	Dynamic 4 × 4 nibble matrices with creation/annihilation configurations and a crossover family, five rounds	128-bit ASCII key	No
S4	MLBPE core [45]	Four-stage multi-level bit-plane permutation with prefix-XOR feedback, driven by a five-dimensional hyperchaotic system	Five real initial values and control parameters	No
S5	ISL-HMC dual S-box [46]	Hyperchaotic ISL-HMC sequences, cube permutation, forward and reverse diffusion, spiral-scanned dual S-boxes, plaintext SHA-512	Real initial values perturbed by the plaintext digest	No
S6	Hoang structure 3 [47]	Five rounds of permutation, whole-image XOR diffusion and AES S-box substitution	ASCII key expanded by SHA-256	No

**Table 2:** Baseline statistics of the six 256 × 256 plaintext images: global Shannon entropy H and adjacent-pixel correlation coefficients in the horizontal ($r_{H}$), vertical ($r_{V}$) and diagonal ($r_{D}$) directions.

Image	Category	H (bits)	$r_{H}$	$r_{V}$	$r_{D}$
Knee MRI	Medical	5.8615	0.9828	0.9905	0.9808
Ankle CT	Medical	3.6465	0.9284	0.9298	0.8701
Head MRI	Medical	3.6448	0.9779	0.9845	0.9645
Cameraman	General	7.0097	0.9335	0.9592	0.9087
Coins	General	6.3162	0.9714	0.9754	0.9605
Rice	General	7.0115	0.9264	0.9432	0.8978

The protocol was identical for all six schemes. Each scheme was given the same plaintext, its context was prepared according to its own specification, and the resulting ciphertext was subjected to the same tests. Plaintext sensitivity was measured over one hundred independent trials per image and per scheme, each trial flipping one uniformly chosen bit at one uniformly chosen pixel position; for the two schemes whose context is derived from the plaintext (S2 and S5) the context was regenerated from the modified plaintext, exactly as those algorithms require. Key sensitivity was measured by flipping a single bit of the secret material — one bit of the 256-bit master key for the proposed scheme, one bit of the ASCII key for S3 and S6, and one bit of the IEEE-754 representation of the first secret real parameter for S4 and S5 — and re-encrypting the unchanged plaintext. Every scheme was verified to recover each of the six plaintexts exactly before any statistic was computed, so all comparisons are between correct implementations. The tables and figures below report values rounded for presentation; the full-precision per-image results underlying every one of them are produced by the three MATLAB programs described in the Methods.

The benchmark convention is fixed once and applied without variation throughout the paper, so that every number in every table is comparable with every other. The same six schemes are evaluated on the same six images at the same 256 × 256 resolution, in one program, with one implementation of each statistic. Local entropy is always computed on non-overlapping 16 × 16 blocks; adjacent-pixel correlation is always computed over all adjacent pairs of the full frame in each of the three directions rather than over a random subsample; NPCR, UACI and the bit-level Hamming distance are always computed from the same set of independent single-bit trials per image and per scheme; the NPCR critical value (99.5693%) and the UACI acceptance interval ([33.2824, 33.6447]%) are always those of a 256 × 256 8-bit image at α = 0.05; the chi-square test always uses 255 degrees of freedom and the 5% critical value 293.25; and the NIST screening always uses the same seven tests with the same parameters at the 1% level. No statistic reported for one scheme is obtained by a route not used for the others, and no value anywhere in this paper is transcribed from a published source. The pass counts of Table 14 are derived from the same underlying results as Tables 5, 8, 9, 11 and 12 and were verified against them cell by cell; in particular the ‘NIST all-7’ column of Table 14 reproduces the final column of Table 12 exactly, a scheme passing all 42 image–test combinations being precisely a scheme that passes all seven tests on all six images.

One qualification belongs with the protocol rather than only with the limitations, because it conditions every comparative statement in this paper. The five reference schemes were re-implemented from their published descriptions, and three of those descriptions leave a step underdetermined: the quantum-operator paper fixes neither the image rotation amount nor the complete crossover table, the MLBPE paper embeds its encryption core inside a detection-and-inpainting pipeline whose front end is out of scope for a full-frame comparison, and the ISL-HMC paper does not state how the four digest-derived quantities are combined into the two initial values. In each case a fixed, reversible resolution was adopted; every resolution is recorded in Table 18 and in the released source code, and a different choice could change the numbers. What is compared here is therefore a faithful re-implementation of each published description together with an explicit resolution of its ambiguities, not the published algorithm as an unambiguous object. Two consequences follow. Results that depend on a resolved detail should be read as conditional on that resolution; results that follow from the structure of an algorithm should not. The failures reported below for the quantum-operator scheme are of the second kind, since a cipher with no chaining and no plaintext-dependent key material cannot have plaintext avalanche whatever crossover table is chosen, and the same holds for the absence of key sensitivity in a scheme whose context is derived from the plaintext digest.

### Theoretical reference values

Several of the statistics used here saturate, so the value a correct cipher should produce can be computed in advance and the measurements interpreted against that prediction rather than against one another. For a 256 × 256 8-bit image, the histogram of an ideal cipher image is a multinomial sample with k = 256 cells and n = 65 536 draws. The plug-in entropy estimator is then biased downwards by approximately $\frac{k\;-\;\text{1}}{\text{2}n\text{ ln }\text{2}}\;=\;\frac{\text{2}\text{5}\text{5}}{\text{2}\;\times \;\text{6}\text{5}\;\text{5}\text{3}\text{6}\;\times \text{ ln }\text{2}}\;\approx \;\text{0}.\text{0}\text{0}\text{2}\text{8}$ bits, so the expected global entropy of a correct scheme is about 7.9972 bits rather than the nominal maximum of 8. The chi-square statistic against the uniform histogram has expectation 255 and standard deviation $\sqrt{\text{5}\text{1}\text{0}}\;\approx \;\text{2}\text{2}.\text{6}$ under the same model, so values roughly in the range 210–300 are the expected outcome and not a mark of quality. Adjacent-pixel correlation computed over the 65 280 adjacent pairs of one direction has a standard error of about $\frac{\text{1}}{\sqrt{\text{6}\text{5}\;\text{2}\text{8}\text{0}}}\;\approx \;\text{0}.\text{0}\text{0}\text{3}\text{9}$ under independence, so any coefficient of magnitude below roughly 0.008 is statistically indistinguishable from zero. For a pair of ciphertexts whose bytes differ independently and uniformly, the expected NPCR is $\text{1}\;-\;{\text{2}}^{-\text{8}}\;=\;\text{9}\text{9}.\text{6}\text{0}\text{9}\text{4}\%$, the expected UACI is 33.4635% and the expected bit-level Hamming distance is 50%.

Read against these predictions, the measurements reported below are what theory requires rather than free-standing achievements. The cipher entropies of the five non-degenerate schemes lie between 7.9967 and 7.9976 bits, straddling the predicted 7.9972; their chi-square statistics lie within a few standard deviations of 255; their mean correlation coefficients are all smaller than 0.006 in magnitude, that is within about one and a half standard errors of zero; and the avalanche figures of the proposed scheme cluster on 99.61% NPCR and 33.45% UACI. This is the precise sense in which these indicators fail to separate designs: a scheme reaches them as soon as its output byte distribution is close to uniform and its avalanche is complete, after which no further improvement exists to be measured. The quantities that do separate the six schemes — whether a one-bit key change alters the ciphertext at all, whether a one-bit plaintext change propagates, and whether a modified ciphertext is detected — are properties of the dependence structure of the algorithm, and each observed failure is therefore explained below by reference to the mechanism responsible for it in the corresponding algorithm, not by the statistic alone.

### Ablation: contribution of the non-standard layers

The final confidentiality and integrity guarantees of the proposed scheme come from AES-256-CTR and HMAC-SHA-256, both standard primitives with their own well-established security arguments. What the Ramanujan switching networks, the Cauchy MDS layers and the two-pass nonlinear feedback diffusion add on top of those primitives is therefore an empirical question, not one that follows automatically from the design. Table 3 reports a four-way ablation intended to isolate that contribution: (i) AES-256-CTR applied directly to the plaintext, with no permutation or diffusion stage and no authentication; (ii) the same AES-CTR output with an HMAC-SHA-256 tag added, isolating the effect of authentication alone; (iii) the Ramanujan switching networks, Cauchy MDS layers and two-pass nonlinear feedback diffusion applied without the outer AES-CTR whitening or HMAC tag, isolating the effect of the non-standard layers alone; and (iv) the complete proposed scheme. Each configuration was evaluated with the same test harness, the same six images and the same statistics used throughout this paper: NPCR and UACI under a one-bit plaintext change, mean adjacent-pixel correlation, and wall-clock encryption time per image.

**Table 3:** Ablation configurations isolating the contribution of the non-standard layers from the standard outer primitives. NPCR and UACI are means over one hundred independent single-bit trials per image and then over the six test images; mean |correlation| is the mean absolute adjacent-pixel correlation over the three directions and the six images; encryption time is, for each image and configuration, the median of ten timed executions of the encryption pipeline on the common test platform described in the Methods, with context construction and key-schedule preparation excluded from the timed region, and the tabulated value is the mean of the six per-image medians. All values were obtained with the harness that produced Tables 2 and 4–14, and the per-image breakdown is produced by the ablation program. Timings in this table are therefore not directly comparable with those of Table 15, which times a full encryption including key and context derivation, discards a warm-up call and takes the mean of ten repeats; under that protocol the complete scheme takes 323.7 ms.

Configuration	NPCR (%)	UACI (%)	Mean |correlation|	Encryption time / image
AES-256-CTR only (no permutation, no diffusion, no authentication)	0.001526	0.000193	0.002674	2.5 ms
AES-256-CTR + HMAC-SHA-256 (authentication added, no permutation, no diffusion)	0.001526	0.000196	0.002674	3.3 ms
Ramanujan switching + Cauchy MDS + two-pass feedback, without outer AES-CTR whitening or HMAC	99.5841	33.4480	0.003232	150.5 ms
Full proposed scheme	99.6074	33.4653	0.003674	148.1 ms

Table 3 reports that ablation. Every configuration is evaluated with the same test harness, the same six images and the same statistics used throughout this paper, and the values quoted are means over the six images. The table separates two questions that the headline comparison merges: how much of the measured behaviour of the proposed scheme is already delivered by AES-256-CTR and HMAC-SHA-256 alone, and what the Ramanujan switching networks, the Cauchy MDS layers and the two-pass nonlinear feedback diffusion contribute once those standard primitives are in place. Configuration (i) is the standard-primitive baseline; configuration (ii) isolates the effect of authentication, which changes no statistic of the ciphertext but changes what a receiver is able to detect; configuration (iii) isolates the non-standard layers by removing the outer whitening and the tag; and configuration (iv) is the complete scheme specified in the Methods.

Four things follow from the table. First, the two standard-primitive configurations have no plaintext diffusion whatever. AES-256-CTR under a fixed nonce is a stream cipher, so a one-bit change in the plaintext alters exactly one byte of the ciphertext and nothing else: the measured NPCR is 0.001526%, which is $\frac{\text{1}}{\text{6}\text{5}\;\text{5}\text{3}\text{6}}$ to every digit reported, and the measured UACI of 0.000193% is the value predicted for a single flipped bit, $\frac{\text{2}\text{5}\text{5}}{\text{8}\;\times \;\text{2}\text{5}\text{5}\;\times \;\text{6}\text{5}\;\text{5}\text{3}\text{6}}\;=\;\text{0}.\text{0}\text{0}\text{0}\text{1}\text{9}\text{1}\%$. This is the same failure mode, and almost the same number, as the 0.0015% reported for the quantum-operator scheme in Table 8. Whatever else the outer whitening contributes, it does not contribute avalanche.

Second, configurations (i) and (ii) are indistinguishable on every ciphertext statistic, as they must be. Their NPCR values agree exactly, their mean absolute correlations agree to every digit reported (0.002674), and their UACI values differ only in the sixth decimal place (0.000193% against 0.000196%), a difference that reflects the magnitude of the single altered byte in each trial and not the presence of the tag. The whole difference between the two rows is that the second can detect a modified ciphertext and the first cannot, and no column of this table registers that. It costs about 0.9 ms per image on the unrounded timings (the displayed values differ by 0.8 ms after rounding), six-tenths of one per cent of the running time of the complete scheme. This is the quantitative form of the central argument of this paper: authentication is invisible to the standard battery, and is exactly the property whose absence matters.

Third, the avalanche is delivered by the non-standard layers alone. Configuration (iii) — the switching networks, the MDS layers and the two-pass feedback, with no outer whitening and no tag — reaches an NPCR of 99.5841% and a UACI of 33.4480%, within 0.025 and 0.016 percentage points of the ideal values 99.6094% and 33.4635% derived above. The complete scheme, configuration (iv), reaches 99.6074% and 33.4653%, which is the ideal to within two parts in a hundred thousand. The gain from (iii) to (iv) is therefore small, as it should be for a keystream exclusive-or applied after mixing is already complete, whereas the gain from (ii) to (iii) is the entire avalanche. The contribution of the non-standard layers beyond AES-CTR is therefore not a matter of degree: without the Ramanujan switching networks, Cauchy MDS layers and two-pass feedback diffusion, the scheme has no plaintext diffusion at all.

Fourth, and this is the argument of the paper turned on its own design, the correlation column separates nothing. The four configurations give mean absolute correlations of 0.002674, 0.002674, 0.003232 and 0.003674, every one of them below the standard error of 0.0039 derived above and therefore indistinguishable from zero. A configuration with no plaintext diffusion whatever and the complete cipher cannot be told apart on this indicator. What we say about published comparisons applies to our own scheme in exactly the same way.

The last column is the price. The non-standard layers raise the time for one 256 × 256 image from 2.5 ms to 148.1 ms, a factor of sixty within this harness, which excludes key-schedule construction and reports the median of ten runs; the corresponding figure under the Table 15 protocol, which includes key and context derivation, is 323.7 ms and 1.62 Mbit/s. The authentication pass accounts for about 0.9 ms of the total on the unrounded timings, the difference between configurations (i) and (ii), which is six-tenths of one per cent. Configurations (iii) and (iv) differ by less than the run-to-run variation of the measurement — 150.5 ms against 148.1 ms, with per-image values overlapping across the whole range — so the whitening and the tag add no time that this harness can resolve on top of the switching and diffusion layers, which account for essentially the entire cost. Whether that exchange is worth making depends on the application, and we report the figures so that a reader can decide rather than be told. One feature of the per-image breakdown behind row (iv) is also worth recording here, because it bears on the plaintext-sensitivity results below: the standard deviation across the hundred trials is close to the theoretical 0.024% on four of the six images, but rises to 0.150% on Rice and 0.356% on the ankle CT slice, which indicates that the shortfall on that image is concentrated in a small number of outlying trials rather than spread uniformly across them.

### Histogram uniformity and global entropy

Table 4 reports the global Shannon entropy of the cipher images. Five of the six schemes cluster in a band of about one thousandth of a bit, from 7.9967 to 7.9976, which is what an 8-bit image of 65 536 pixels with an essentially uniform histogram should give; the theoretical maximum of 8 bits is unattainable at this sample size, and the small deficit is the sampling noise of a multinomial with 256 cells and 65 536 draws. On this indicator the proposed scheme, the fuzzy scheme, the MLBPE core, the ISL-HMC scheme and the Hoang structure are indistinguishable. Any ranking among them would be a ranking of rounding noise. These values are exactly what the multinomial argument above predicts: the five clustered schemes differ from the predicted 7.9972 bits by at most $\text{5}\;\times \;{\text{1}\text{0}}^{-\text{4}}$ bits, which is of the same order as the sampling fluctuation of the estimator itself, so the ordering among them is an ordering of noise and carries no information about the ciphers.

**Table 4:** Global Shannon entropy (bits) of the cipher images. The last row is the mean over the six images.

Image	Proposed	S2 Fuzzy [43]	S3 Quantum [44]	S4 MLBPE [45]	S5 ISL-HMC [46]	S6 Hoang-3 [47]
Knee MRI	7.9970	7.9973	7.6711	7.9976	7.9972	7.9974
Ankle CT	7.9970	7.9969	6.9320	7.9967	7.9973	7.9972
Head MRI	7.9970	7.9970	7.1846	7.9972	7.9967	7.9970
Cameraman	7.9969	7.9971	7.9678	7.9970	7.9973	7.9971
Coins	7.9974	7.9972	7.9295	7.9972	7.9968	7.9974
Rice	7.9975	7.9972	7.9707	7.9969	7.9968	7.9974
Mean	7.9971	7.9971	7.6093	7.9971	7.9970	7.9972

The quantum-operator scheme is the exception, and the exception is large. Its cipher entropy falls to 6.9320 bits on the ankle CT slice and 7.1846 bits on the head MRI slice, and it never exceeds 7.9707 bits on any image. The pattern is diagnostic: the entropy deficit tracks the redundancy of the plaintext almost exactly, being worst on the two lowest-entropy medical images and mildest on Cameraman and Rice. A cipher whose output entropy depends on its input entropy is not producing a uniform distribution; it is producing a re-labelled version of the input distribution. Fig 2 shows the effect directly.

**Fig 2:**
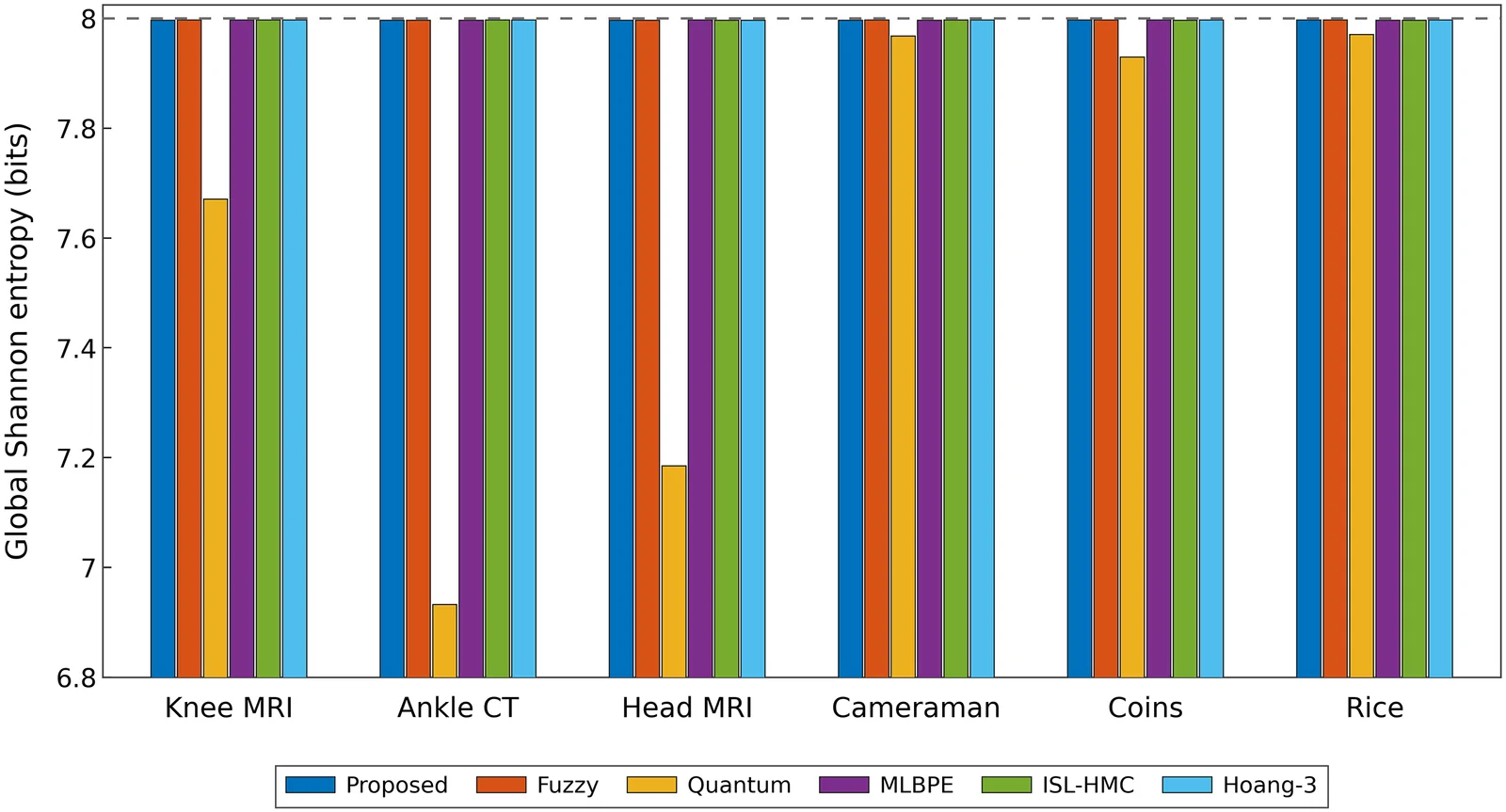
Global Shannon entropy of the cipher images for the six schemes and six test images. The dashed line marks the theoretical maximum of 8 bits. Five schemes are visually indistinguishable at this scale; the quantum-operator scheme loses between 0.03 and 1.07 bits depending on the redundancy of the plaintext.

The chi-square goodness-of-fit statistic against a uniform histogram, reported in Table 5, sharpens the picture. With 255 degrees of freedom the 5% critical value is 293.25. The proposed scheme, the fuzzy scheme and the Hoang structure stay below that threshold on all six images; the MLBPE core exceeds it once (298.91 on the ankle CT slice) and the ISL-HMC scheme exceeds it once (300.13 on the head MRI slice). One exceedance in six is exactly what a 5% test is supposed to produce on roughly one occasion in twenty per image, so with six images per scheme these single failures carry no evidential weight on their own. The quantum-operator scheme, by contrast, produces statistics of 2 687–168 296, between one and three orders of magnitude above the critical value, and a p-value indistinguishable from zero on every image. Its histogram is not merely imperfect; it is visibly non-uniform.

**Table 5:** Chi-square goodness-of-fit statistic of the cipher histograms against the uniform distribution (255 degrees of freedom; 5% critical value 293.25).

Image	Proposed	S2 Fuzzy [43]	S3 Quantum [44]	S4 MLBPE [45]	S5 ISL-HMC [46]	S6 Hoang-3 [47]
Knee MRI	270.86	245.46	42141.21	220.33	256.51	239.45
Ankle CT	271.37	283.78	168296.41	298.91	247.09	256.00
Head MRI	273.77	272.18	117161.07	251.47	300.13	275.66
Cameraman	284.25	261.02	2867.57	273.93	240.12	259.10
Coins	236.80	252.70	6558.40	252.25	293.18	235.51
Rice	229.84	257.38	2687.11	278.16	289.55	237.44
Mean	261.15	262.09	56618.63	262.51	271.10	250.53

### Local entropy and structural residue

Global entropy is a weak test because it is blind to spatial arrangement: a cipher image assembled from perfectly uniform but locally structured blocks would score 8 bits. Table 6 therefore reports the local Shannon entropy computed on non-overlapping 16 × 16 blocks, together with the plaintext-to-ciphertext PSNR and SSIM. Five schemes give a block-mean of about 7.173 to 7.176 bits with a standard deviation near 0.05 and a worst block no lower than 6.96, which is the distribution expected from 256 uniform draws over 256 levels. The quantum-operator scheme gives a block-mean of 6.177 with a standard deviation fifteen times larger and a worst block of 3.95 bits, meaning that entire regions of its output remain almost as structured as the plaintext.

**Table 6:** Local Shannon entropy on 16 × 16 blocks (mean over blocks and images, mean block-to-block standard deviation, and the lowest single block observed across all six images) with plaintext-to-ciphertext PSNR and SSIM.

Scheme	Local H mean	Local H s.d.	Local H min	PSNR (dB)	SSIM
Proposed	7.1744	0.0527	6.9831	7.404	0.0340
S2 Fuzzy [43]	7.1737	0.0510	6.9894	7.397	0.0339
S3 Quantum [44]	6.1774	0.7831	3.9528	7.566	0.0344
S4 MLBPE [45]	7.1763	0.0528	6.9667	7.396	0.0338
S5 ISL-HMC [46]	7.1751	0.0532	6.9911	7.399	0.0337
S6 Hoang-3 [47]	7.1729	0.0516	7.0112	7.398	0.0338

The PSNR and SSIM columns behave as expected and are reported for completeness rather than for discrimination. All six schemes place the cipher between 7.39 and 7.57 dB from the plaintext with an SSIM of 0.034, values that indicate no perceptual relationship whatsoever. The quantum-operator scheme in fact has the highest PSNR of the group, which illustrates why plaintext-to-ciphertext distortion measures are of essentially no use in security assessment: the scheme that retains the most structure also scores best on the indicator that is supposed to measure how thoroughly structure was destroyed.

### Adjacent-pixel correlation

Table 7 summarises the correlation coefficients between adjacent pixel pairs of the cipher images in the three principal directions, averaged over the six images, together with the largest absolute value observed in any single image and direction. Fig 3 plots the same data as mean absolute coefficients by direction on a logarithmic scale, which separates the five clustered schemes from the quantum-operator scheme more clearly than the tabulated signed means do. With 65 280 pairs per direction, the standard error of a sample correlation under the null hypothesis of independence is about 0.0039, so any coefficient below roughly 0.008 in absolute value is within two standard errors of zero. The proposed scheme, the fuzzy scheme, the MLBPE core, the ISL-HMC scheme and the Hoang structure all satisfy that in every direction, and the honest reading of this table is that these five schemes are statistically equivalent: their maximum absolute coefficients across all eighteen image–direction combinations lie between 0.0082 and 0.0123, with no scheme uniformly smallest across all three directions. Measured against the standard error of 0.0039 derived above, the mean coefficients of the five non-degenerate schemes fall within about one and a half standard errors of zero in every direction, so their residual correlation is not distinguishable from that of an independent uniform sequence of the same length. The structural reason the proposed scheme reaches this level is the alternation of positional and value mixing: the switching networks carry the eight bytes of an MDS block to widely separated positions, and the next MDS layer, whose branch number is nine, spreads each of them over a fresh block, so no pair of adjacent output positions derives from a pair of adjacent input positions.

**Table 7:** Adjacent-pixel correlation of the cipher images. The first three columns are signed means over the six images; the last column is the largest absolute coefficient observed in any image and direction.

Scheme	Horizontal	Vertical	Diagonal	max |r|
Proposed	−0.00013	0.00181	0.00281	0.01021
S2 Fuzzy [43]	−0.00229	0.00113	−0.00040	0.01219
S3 Quantum [44]	−0.03761	−0.01857	−0.00873	0.13198
S4 MLBPE [45]	0.00018	0.00097	0.00148	0.00816
S5 ISL-HMC [46]	0.00161	0.00236	−0.00056	0.00815
S6 Hoang-3 [47]	−0.00057	−0.00083	0.00333	0.01228

**Fig 3:**
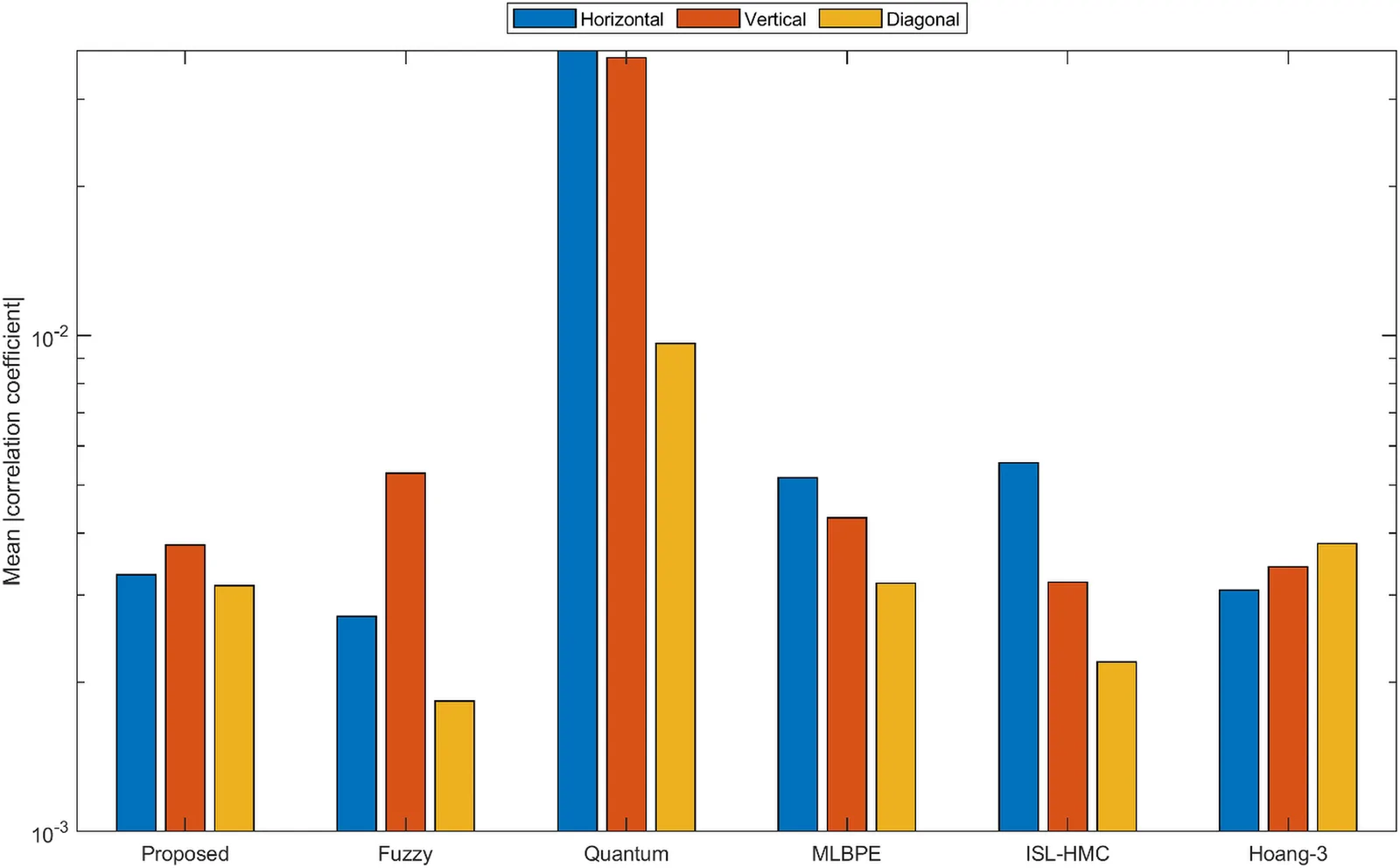
Mean absolute adjacent-pixel correlation of the cipher images by direction, on a logarithmic scale. Five schemes lie below 0.006; the quantum-operator scheme is an order of magnitude worse in the horizontal and vertical directions.

The quantum-operator scheme again separates itself, with a mean absolute horizontal coefficient of 0.0376 and a single vertical coefficient of −0.1320 on the ankle CT slice. A vertical coefficient of −0.13 is more than thirty standard errors from zero, and even the horizontal mean of 0.0376 is about ten. That cannot be put down to sampling error: some of the plaintext's spatial structure has survived encryption, which is what the entropy and histogram figures for this scheme already suggested.

### Plaintext sensitivity

Differential behaviour is where the schemes stop agreeing. Tables 8 and 9 report the mean NPCR and UACI over one hundred single-bit plaintext trials per image, and Fig 4 plots them against the standard decision thresholds. For a 256 × 256 8-bit image the NPCR critical value at the 5% level is 99.5693%, and the two-sided UACI acceptance interval at the same level is [33.2824, 33.6447]%. The perturbation used throughout this subsection is a single bit at a single pixel, which is the hardest case for a cipher to propagate and the one the standard NPCR and UACI thresholds are derived for; multi-bit and multi-pixel perturbations are reported separately below, since they test a different property.

**Table 8:** NPCR (%) under a one-bit plaintext change, averaged over one hundred independent trials per image; the final row is the mean over the six images. The single-trial 5% critical value is 99.5693%. Table 14 records the conventional decision, the tabulated mean against that value, so that every scheme is judged by the rule its own paper would use; the trial-level statistics that carry the real information are reported in the text below and produced by the Tables 15–17 program.

Image	Proposed	S2 Fuzzy [43]	S3 Quantum [44]	S4 MLBPE [45]	S5 ISL-HMC [46]	S6 Hoang-3 [47]
Knee MRI	99.6080	99.5987	0.0015	93.3218	99.6124	99.6083
Ankle CT	99.6093	99.6050	0.0015	93.3569	99.6104	99.6116
Head MRI	99.6132	99.5856	0.0015	93.3291	99.6092	99.6112
Cameraman	99.6142	99.6118	0.0015	93.3201	99.6056	99.6114
Coins	99.6163	99.6070	0.0015	93.3191	99.6107	99.6089
Rice	99.5847	99.5988	0.0015	93.3366	99.6127	99.6083
Mean	99.6076	99.6012	0.0015	93.3306	99.6102	99.6099

**Table 9:** UACI (%) under a one-bit plaintext change, averaged over one hundred independent trials per image; the final row is the mean over the six images. The single-trial 5% acceptance interval is [33.2824, 33.6447]%, applied as for NPCR.

Image	Proposed	S2 Fuzzy [43]	S3 Quantum [44]	S4 MLBPE [45]	S5 ISL-HMC [46]	S6 Hoang-3 [47]
Knee MRI	33.4343	33.4710	0.0003	30.1460	33.4323	33.4775
Ankle CT	33.4288	33.4805	0.0003	30.1255	33.4857	33.4363
Head MRI	33.4156	33.4564	0.0003	30.0847	33.5407	33.4847
Cameraman	33.4651	33.4554	0.0003	30.0995	33.5035	33.4424
Coins	33.4769	33.5074	0.0003	30.0717	33.4491	33.4646
Rice	33.4619	33.4685	0.0003	30.1690	33.4803	33.5049
Mean	33.4471	33.4732	0.0003	30.1161	33.4819	33.4684

**Fig 4:**
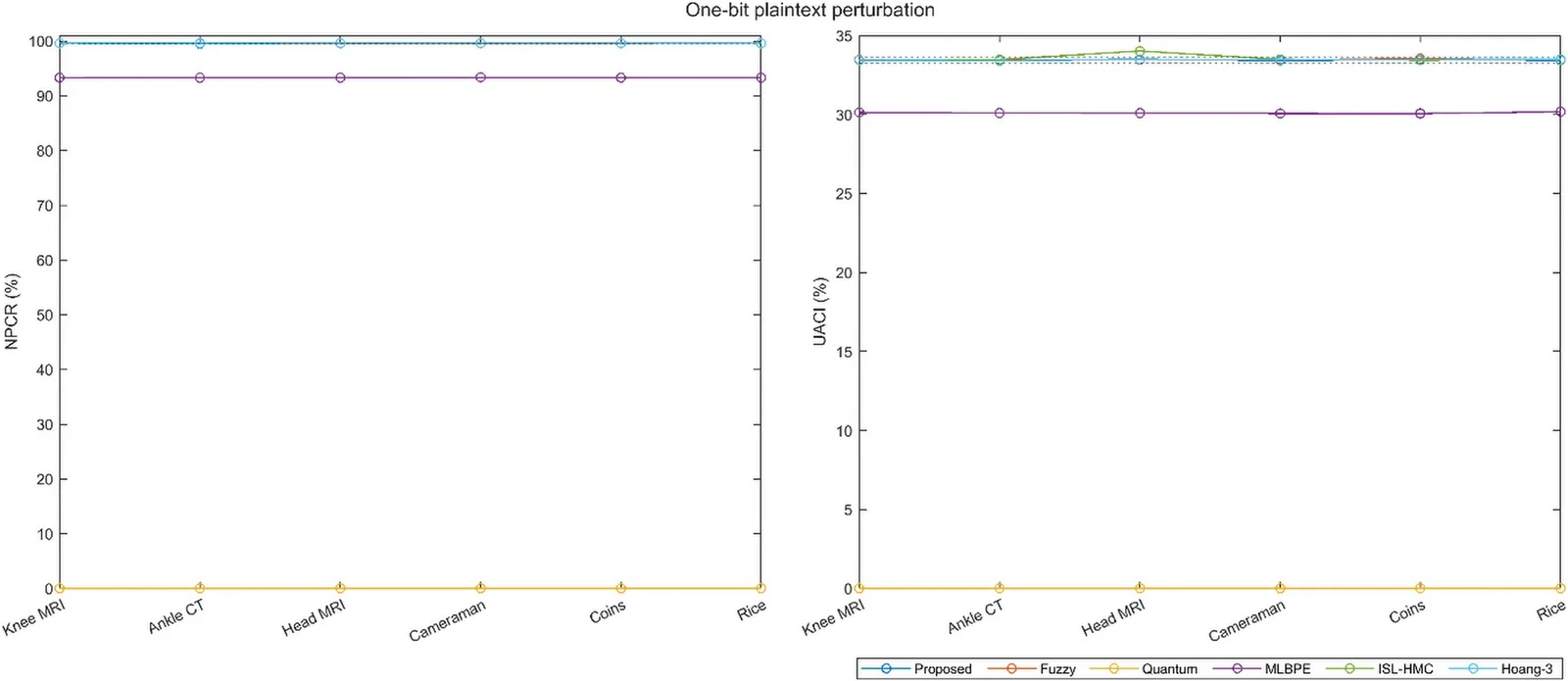
NPCR (left) and UACI (right) under one-bit plaintext perturbation. Dashed lines mark the 5% NPCR critical value and the two-sided UACI acceptance interval. Four schemes overlap on the thresholds; the MLBPE core sits well below both, and the quantum-operator scheme is effectively at zero. The figure has been regenerated from the hundred-trial run: each marker is the mean over one hundred independent single-bit trials for that scheme and image, and the vertical bars show ± one standard deviation across those trials, so that the stability of a scheme’s differential behaviour can be read off alongside its mean.

Those statistics are more discriminating than the means, and they reveal a difference the means conceal. For each image and scheme we record the standard deviation across the hundred trials, the smallest NPCR observed in any single trial, and the number of individual trials whose NPCR exceeds the single-trial critical value. An ideal cipher produces about ninety-five such passes in a hundred, since the critical value is the 5% quantile of the single-trial distribution. The proposed scheme records between 93 and 97 passes across the six images, the ISL-HMC scheme between 91 and 98, and the Hoang structure between 92 and 97; all three are consistent with the ideal rate. The fuzzy scheme records between 64 and 88, and on the head MRI slice only 64, which is far below what an ideal cipher would give and is not visible in its mean of 99.5856% on that image. The MLBPE core and the quantum-operator scheme record none at all on any image. On this reading the fuzzy scheme joins the list of designs whose differential behaviour is weaker than its headline numbers suggest, and it does so on evidence that a table of means cannot carry.

The trial-level view is also where our own scheme stops looking uniformly good, and we record that rather than leave it to a reader to find. Its per-trial standard deviation ranges from 0.0255% on Coins to 0.2785% on Rice, against a theoretical 0.0244% for an ideal cipher, and its per-trial minimum falls to 98.8724% on the knee MRI slice and 96.8460% on Rice. The ISL-HMC scheme and the Hoang structure are tighter, with standard deviations between 0.0217% and 0.0307% on every image and minima no lower than 99.5255%. The proposed scheme therefore has a small number of trials in which a flipped bit propagates less completely than in the rest, which is invisible in a mean of 99.6076% and is the sort of thing the ablation of Table 3 would locate if it recurred. We do not have an explanation for it that we are willing to assert, and we flag it as the one place where the trial-level data are less flattering to our design than to two of the reference schemes.

Four schemes — the proposed one, the fuzzy scheme, the ISL-HMC scheme and the Hoang structure — cluster tightly around the ideal values, with NPCR between 99.585% and 99.616% and UACI between 33.42% and 33.54%, and with mean bit-level Hamming distances of 50.00%, 49.96%, 50.02% and 50.01% respectively, all within a twentieth of a percentage point of the ideal one half. On the means, all four pass on all six images. That agreement is the point of the study rather than a result in it: on these two indicators the four designs are interchangeable, and the two that fail do so by margins so large that no threshold refinement would alter the verdict.

The MLBPE core is a clear outlier in a way that is easy to misread. Its NPCR is 93.33% and its UACI is 30.11%, both far outside the acceptance regions, and yet its bit-change rate is 50.00%, at the ideal one half. The two facts are consistent: the four-stage bit-plane architecture spreads a single input bit almost uniformly at the bit level, but the final repacking maps bits back into bytes in a way that leaves about one byte in fifteen unchanged. In other words the scheme achieves near-complete bit-level avalanche and incomplete byte-level avalanche. Whether this is a security problem depends on the attack model, but it is a measurable and repeatable structural signature, and it is invisible to anyone who reports only bit-level statistics.

The quantum-operator scheme fails outright. Its NPCR is 0.0015%, which corresponds to a single changed byte out of 65 536, and its UACI is 0.0003%. The reason is structural rather than incidental: the cipher applies a fixed sequence of nibble substitutions and rotations whose key material is derived from the ASCII key alone, with no plaintext dependence and no chaining. A one-bit change in the plaintext therefore changes one nibble, that nibble is substituted, and nothing else moves. Under chosen-plaintext analysis this position-wise separability is a serious structural weakness, because each output position depends on exactly one input position and the affected positions can therefore be studied independently rather than through a globally diffusive mapping.

The hundred-trial run also settles a question raised by the thirty-trial version of this experiment, in which the proposed scheme returned a mean NPCR of 99.5189% on the ankle CT slice, below the 5% critical value. That shortfall does not reproduce. At one hundred trials the ankle CT mean is 99.6093%, within two parts in a hundred thousand of the ideal 99.6094%, and the earlier figure is explained as a small-sample artefact rather than a structural property of the image. We also record why the statistical treatment in the submitted version was wrong, since the correction matters beyond this one cell. The critical value of equation (29) is derived for a single pair of ciphertexts, whereas the quantity tabulated here is a mean over independent trials, whose standard error is smaller by a factor of the square root of the number of trials; comparing a trial mean with a single-trial threshold is not a valid significance test, and describing the result as ordinary false rejection was not supportable. Table 14 nonetheless records the conventional mean-based decision, because that is the rule under which each of the compared schemes was judged in its own paper and changing it would break the comparison this study exists to make. What we do instead is report the trial-level statistics alongside it.

### Key and secret-parameter sensitivity

The one-bit key sensitivity results, in Table 10 and Fig 5, are the most consequential of the study, because a cipher whose output does not change when its key changes offers no confidentiality at all regardless of how uniform its ciphertext looks.

**Table 10:** Sensitivity of the ciphertext to a one-bit change in the secret material, averaged over the six images. The final column counts the images on which NPCR exceeded the 5% critical value of 99.5693%.

Scheme	NPCR (%)	UACI (%)	Bit HD (%)	Images passing
Proposed	99.6188	33.4390	49.9892	6/6
S2 Fuzzy [43]	0.0000	0.0000	0.0000	0/6
S3 Quantum [44]	97.0802	29.4818	47.1205	0/6
S4 MLBPE [45]	0.0000	0.0000	0.0000	0/6
S5 ISL-HMC [46]	49.8062	16.7089	24.9909	3/6
S6 Hoang-3 [47]	99.6211	33.4560	49.9775	6/6

**Fig 5:**
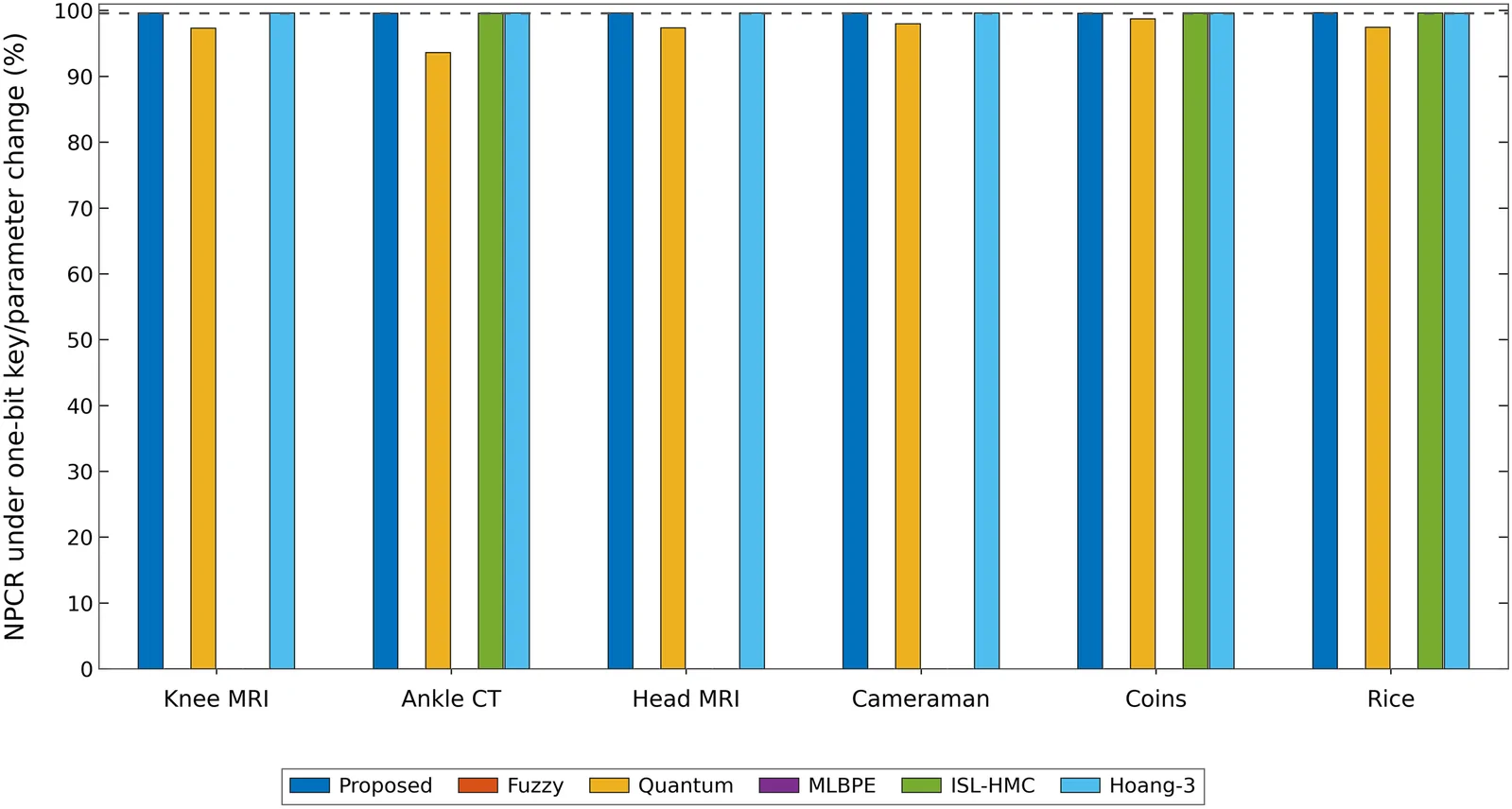
NPCR under a one-bit change of the secret key or secret parameter. The dashed line is the 5% critical value. The fuzzy scheme and the MLBPE core produce identical ciphertexts before and after the change on every image; the ISL-HMC scheme does so on three of six.

Two schemes produced bit-for-bit identical ciphertexts before and after the key change, on every image: NPCR, UACI and Hamming distance are all exactly zero. For the fuzzy scheme the reason is by construction — in the configuration described in its source publication, the encryption context is derived from the SHA-512 digest of the plaintext and the key text is not mixed into it, so the key is not a key at all but a formal parameter. For the MLBPE core the reason is numerical: its secret material consists of five double-precision initial values for a five-dimensional continuous hyperchaotic system, and flipping the least significant bit of the first of them, a relative perturbation of about $\text{2}\;\times \;{\text{1}\text{0}}^{-\text{1}\text{6}}$, produced sequences that were identical after quantisation to the byte and permutation ranges used by the algorithm. The system is chaotic in the mathematical sense, but the finite-precision integration with a fixed step of 0.01 followed by quantisation absorbs the perturbation. This is a well-known hazard of chaos-based key schedules and it is the reason cryptographic key schedules use hash functions rather than differential equations.

**Table 11:** Per-image NPCR (%) under a one-bit change of the secret material.

Image	Proposed	S2 Fuzzy [43]	S3 Quantum [44]	S4 MLBPE [45]	S5 ISL-HMC [46]	S6 Hoang-3 [47]
Knee MRI	99.6292	0.0000	97.3221	0.0000	0.0000	99.6216
Ankle CT	99.5911	0.0000	93.6310	0.0000	99.5850	99.6399
Head MRI	99.6384	0.0000	97.3541	0.0000	0.0000	99.6445
Cameraman	99.6185	0.0000	97.9889	0.0000	0.0000	99.6155
Coins	99.5880	0.0000	98.7350	0.0000	99.6399	99.6124
Rice	99.6475	0.0000	97.4503	0.0000	99.6124	99.5926

The ISL-HMC scheme is intermediate and unstable. On three of the six images (ankle CT, Coins, Rice; Table 11) a one-bit change of the base value $x_{\text{0}}$ produced full avalanche, with NPCR near 99.6%; on the other three (knee MRI, head MRI, Cameraman) it produced no change at all. The scheme derives its actual initial conditions by adding plaintext-digest quantities to the base values and reducing modulo one, so whether a one-ULP perturbation of the base survives depends on the digest, and therefore on the image. A key sensitivity that is a coin flip conditioned on the plaintext is not a usable security property.

The quantum-operator scheme responds to key changes, with NPCR between 93.63% and 98.74%, but never reaches the critical value on any image and shows a UACI of 29.48% on average, well below the acceptance interval. The proposed scheme and the Hoang structure both give full key avalanche on every one of the six images, with mean NPCR of 99.6188% and 99.6211% and mean bit-level Hamming distances of 49.99% and 49.98% respectively; on this indicator the two are indistinguishable.

### Randomness screening

Seven tests from the NIST SP 800−22 suite were applied to the 524 288-bit sequence obtained by unpacking each cipher image most-significant-bit first: frequency, block frequency with M = 128, runs, spectral (DFT), approximate entropy with m = 10, and the two serial statistics with m = 3. This is a screening subset rather than the full suite, applied to a single sequence per image, so it is used here to detect gross departures from randomness rather than to certify a generator. Table 12 summarises the corresponding pass counts, while Fig 6 shows the image-by-test pass/fail pattern for each scheme; the individual p-values for all 252 image–scheme–test combinations are written out by the six-scheme comparison program.

**Table 12:** NIST SP 800−22 screening results: number of images (out of six) whose cipher passed each test at the 1% level, the total over all 42 image–test combinations, and the number of images passing all seven tests simultaneously. These are screening outcomes on a single sequence per image, not a certification of randomness.

Scheme	Frequency	Block freq.	Runs	Spectral (DFT)	Approx. entropy	Serial I	Serial II	Total	All 7
Proposed	6/6	6/6	6/6	6/6	6/6	6/6	6/6	42/42	6/6
S2 Fuzzy [43]	6/6	6/6	6/6	5/6	6/6	6/6	6/6	41/42	5/6
S3 Quantum [44]	3/6	6/6	0/6	0/6	0/6	0/6	1/6	10/42	0/6
S4 MLBPE [45]	6/6	6/6	6/6	6/6	6/6	6/6	6/6	42/42	6/6
S5 ISL-HMC [46]	6/6	6/6	6/6	6/6	6/6	6/6	6/6	42/42	6/6
S6 Hoang-3 [47]	6/6	6/6	6/6	5/6	5/6	6/6	6/6	40/42	4/6

**Fig 6:**
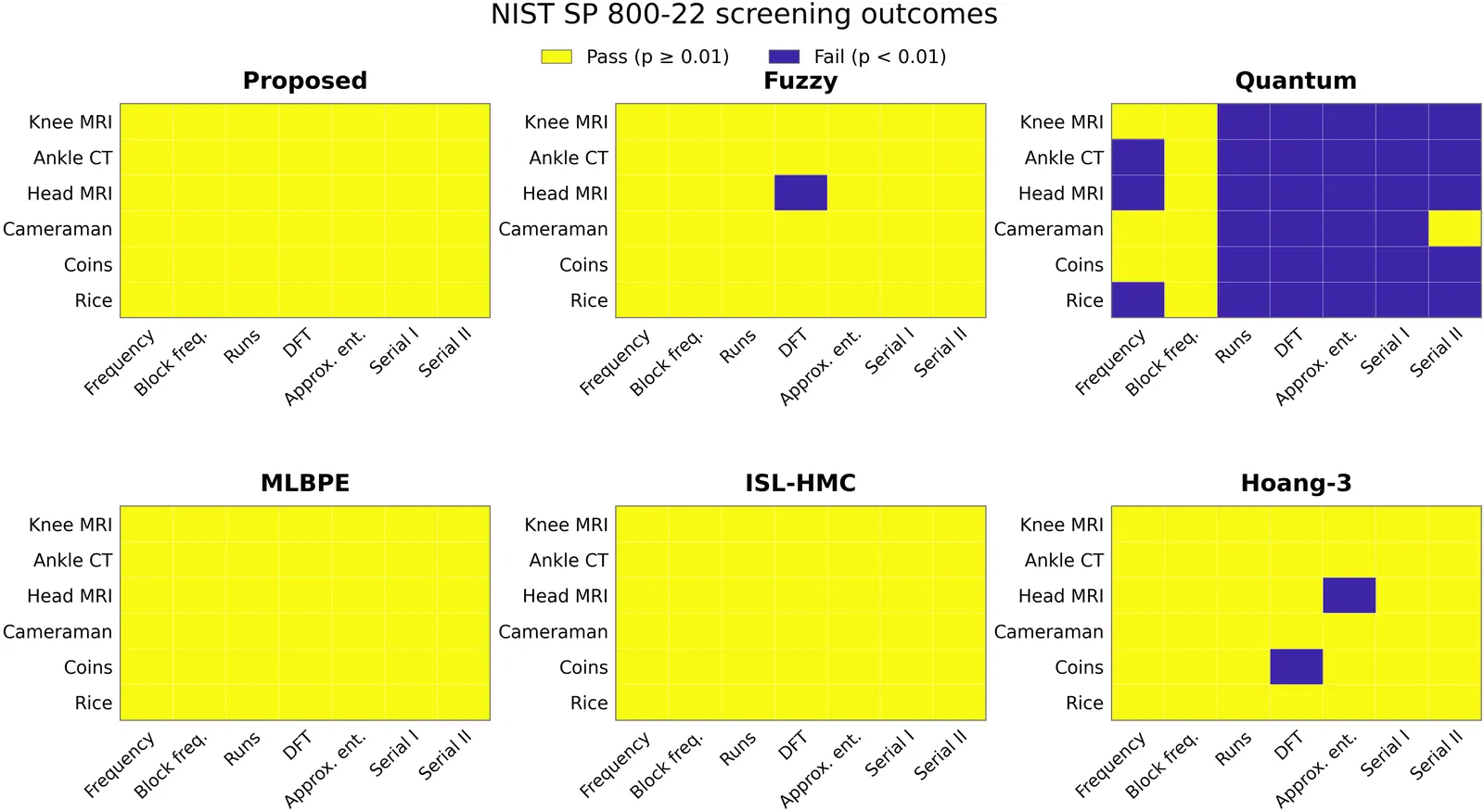
NIST SP 800−22 screening outcomes for the six encryption schemes across the six test images. Each cell indicates the outcome of one of seven screening tests at the 1% significance level (p ≥ 0.01): yellow denotes a pass and purple denotes a failure. The proposed scheme, MLBPE and ISL-HMC pass all 42 image–test combinations, whereas the quantum-operator scheme shows systematic failures.

As shown in Fig 6, the proposed scheme, the MLBPE core and the ISL-HMC scheme pass all 42 combinations. The fuzzy scheme fails one and the Hoang structure fails two. With 42 tests per scheme at a 1% level, the expected number of false rejections is 0.42, so one or two failures per scheme is a slightly elevated but unremarkable count and none of the individual failures recurs across images or clusters within a scheme. The fuzzy scheme’s single failure is the spectral (DFT) test on the head MRI slice; the Hoang structure’s two failures are the approximate entropy test on the head MRI slice and the spectral test on Coins.

Because a pass count alone does not identify the image responsible for a failure, the failing combinations are stated explicitly. Excluding the quantum-operator scheme, whose failures are spread across all six images, three isolated failures occur in the whole comparison: the spectral (DFT) test of the fuzzy scheme on the head MRI slice, the approximate entropy test of the Hoang structure on the same image, and the spectral test of the Hoang structure on Coins. The approximate entropy column of Table 12 therefore shows 5/6 for the Hoang structure: only one image, the head MRI slice, fails the test at the 1% level. The complete numerical p-values for all 252 image–scheme–test combinations are written out by the six-scheme comparison program, and Fig 6 presents the corresponding pass/fail pattern, so that any individual cell of Table 12 can be traced to the image responsible for it.

The relation between Table 12 and Table 14 is also worth stating explicitly, since the two report different quantities and a reader comparing them directly may expect them to agree numerically. The ‘Total’ column of Table 12 counts image–test combinations out of 42; the ‘All 7’ column of Table 12 and the ‘NIST all-7’ column of Table 14 count images out of six on which every one of the seven tests passes simultaneously, and these two columns are identical by construction. The apparent discrepancies therefore resolve as follows. The ISL-HMC scheme passes 42 of 42 combinations, so all six images pass all seven tests and both columns read 6/6. The Hoang structure fails two combinations out of 42, giving 40/42; because those two failures fall on two different images — approximate entropy on the head MRI slice and the spectral test on Coins — two images fail to pass all seven tests and the count of images is 4/6, not 5/6. Two failures reduce the image count by two only when they are on different images, which is the case here. We have verified every cell of Table 14 against the underlying results in this way.

The quantum-operator scheme fails 32 of 42 combinations, including every instance of the runs, spectral and approximate-entropy tests, with p-values that underflow to zero. Several of its serial p-values are of order ${\text{1}\text{0}}^{-\text{3}\text{0}\text{0}}$. This is not a marginal statistical result; the bit sequence is not remotely random.

The status of this exercise should be stated without ambiguity: it is a coarse screen, not a certification of randomness, and no claim of the latter is made anywhere in this paper. Seven of the fifteen tests of SP 800−22 were applied to one 524 288-bit sequence per image at the 1% level. A certification in the sense intended by the specification requires the full suite, sequences of at least ${\text{1}\text{0}}^{\text{6}}$ bits, at least 55 independent sequences per generator so that the proportion of passing sequences can be compared with its confidence interval, and a second-level analysis testing the uniformity of the p-values themselves; none of those conditions is met here, and with a single sequence per image the outcome of any individual test is a Bernoulli draw whose variance is too large to support a ranking. What the screen can do, and all it is used for here, is expose gross departures from randomness, and it does so decisively in one case: a scheme failing 32 of 42 combinations with p-values that underflow to zero is not failing by chance, whereas the one and two isolated failures of the fuzzy scheme and the Hoang structure are within the range expected from 42 tests at the 1% level and are treated here as carrying no evidential weight. Accordingly, the ‘NIST all-7’ column of Table 14 should be read as a screening outcome and not as a certificate, and a design intended for deployment should be evaluated with the complete suite at the recommended sample sizes, as stated in the Limitations.

### Integrity, tamper detection and key rejection

The proposed scheme computes an HMAC-SHA-256 tag over the header, the nonce and the ciphertext, and its decryption routine verifies that tag with a constant-time comparison before performing any inverse transformation. Three rejection experiments were run on every image: flipping a single bit of the transmitted ciphertext, flipping one bit in each of a randomly chosen 1% of ciphertext bytes, and decrypting with a master key differing in one bit. All three were rejected on all six images, in every case, with no false rejection of an untampered ciphertext.

None of the five reference schemes provides an authentication or rejection interface. This is stated as a property of the published algorithms rather than as a deficiency of our implementations: we deliberately did not add a tag to them, because doing so would have changed the algorithm being measured. The practical consequence is reported in Table 13. A receiver using any of the five reference schemes cannot distinguish a legitimate ciphertext from one that has been modified in transit, and cannot detect that it has used the wrong key; it simply produces a different picture. For the medical images in this study that is a clinically relevant failure mode.

**Table 13:** Integrity behaviour. “n/a” indicates that the published algorithm provides no authentication interface, so the corresponding experiment cannot be performed without altering the scheme.

Scheme	Exact recovery	Authenticated	1-bit tamper rejected	1% tamper rejected	Wrong key rejected
Proposed	6/6	Yes	6/6	6/6	6/6
S2 Fuzzy [43]	6/6	No	n/a	n/a	n/a
S3 Quantum [44]	6/6	No	n/a	n/a	n/a
S4 MLBPE [45]	6/6	No	n/a	n/a	n/a
S5 ISL-HMC [46]	6/6	No	n/a	n/a	n/a
S6 Hoang-3 [47]	6/6	No	n/a	n/a	n/a

Because the proposed scheme draws a fresh 96-bit nonce for every encryption, encrypting the same plaintext twice under the same key yields unrelated ciphertexts; measured over the six images, a nonce change alone gives a mean NPCR of 99.606%, a mean UACI of 33.461% and a mean bit-level Hamming distance of 50.034%, indistinguishable from the plaintext and key avalanche figures. None of the five reference schemes is randomised, so all of them are deterministic functions of the plaintext and are therefore vulnerable to the standard traffic-analysis consequence: identical frames encrypt to identical ciphertexts, and a change between two frames is visible as a change in the ciphertext.

### Overall comparison

Table 14 collects the pass counts across the five decision-based tests. Read row by row, it makes the central point of the study. On the two indicators that appear in nearly every image-encryption paper — histogram uniformity and correlation — five of the six schemes are equivalent and would all be described as excellent. On plaintext sensitivity one scheme collapses and another fails by a wide and structurally explicable margin. On key sensitivity two schemes fail completely and one fails on half of the images. On integrity, five of six schemes have nothing to offer. A comparison restricted to the conventional battery would have reported six broadly equivalent ciphers.

**Table 14:** Number of images (out of six) passing each decision test: chi-square uniformity at 5%, plaintext NPCR at 5%, plaintext UACI at 5%, key-change NPCR at 5%, and all seven NIST screening tests at 1%. For plaintext NPCR and UACI, Table 14 retains the conventional literature comparison, in which the tabulated trial mean is compared with the standard single-trial reference limits; as explained in the Methods this is kept for comparability and is not treated as a formal significance test of an averaged quantity, and the trial-level pass counts and variability are reported separately in the text. The final column reports the screening outcome of Table 12 and is not a certification of randomness.

Scheme	Chi-square	NPCR (plain)	UACI (plain)	NPCR (key)	NIST all-7
Proposed	6/6	6/6	6/6	6/6	6/6
S2 Fuzzy [43]	6/6	6/6	6/6	0/6	5/6
S3 Quantum [44]	0/6	0/6	0/6	0/6	0/6
S4 MLBPE [45]	5/6	0/6	0/6	0/6	6/6
S5 ISL-HMC [46]	5/6	6/6	6/6	3/6	6/6
S6 Hoang-3 [47]	6/6	6/6	6/6	6/6	4/6

### Additional evaluation indexes and computational cost

The battery reported above covers histogram uniformity, global and local entropy, adjacent-pixel correlation, plaintext and key differential behaviour at both byte and bit level, randomness screening, integrity, and plaintext-to-ciphertext PSNR and SSIM. So that the comparison is not confined to the indicators that happen to favour any particular design, Table 15 adds four further indexes that are common in this literature and are computed here, as everywhere else in the paper, by one implementation applied identically to all six schemes: the mean squared error between plaintext and ciphertext, the variance of the cipher histogram, the key space, and the wall-clock encryption time with the corresponding throughput on the common test platform.

**Table 15:** Additional evaluation indexes. MSE and histogram variance are means over the six test images; the time column is the mean wall-clock encryption time for one 256 × 256 image on the common test platform described in the Methods (MATLAB R2020a, Intel Core i5-6200U at 2.30 GHz, 8 GB RAM, 64-bit Windows 10) and the final column is the corresponding throughput in Mbit/s.

Scheme	Mean MSE	Histogram variance	Key space	Time (s/image)	Mbit/s
Proposed	12478.2	245.9	${\text{2}}^{\text{2}\text{5}\text{6}}$ key + 96-bit nonce	0.3237	1.62
S2 Fuzzy [43]	12461.4	262.1	No effective secret key	0.0514	10.19
S3 Quantum [44]	11847.4	56618.6	${\text{2}}^{\text{1}\text{2}\text{8}}$	0.0624	8.40
S4 MLBPE [45]	12463.6	262.5	Five double parameters	3.5901	0.146
S5 ISL-HMC [46]	12461.0	271.1	Real parameters + digest	1.6226	0.323
S6 Hoang-3 [47]	12458.3	250.5	$\leq \;{\text{2}}^{\text{2}\text{5}\text{6}}$ (key text)	4.0094	0.131

Two remarks apply to these indexes, and the measured values bear both out. Mean squared error and histogram variance are functions of the same two distributions already summarised by PSNR and by the chi-square statistic, so they are reported for comparability with the literature rather than as independent evidence: a scheme with a near-uniform cipher histogram necessarily has a small histogram variance and a large plaintext-to-ciphertext MSE, and neither quantity can distinguish a cipher with full key avalanche from one with none. That is what happens here. The mean squared error lies between 12 458 and 12 478 for five of the six schemes, a spread of 0.16%, and the histogram variance lies between 246 and 271 against the value 255 that a uniform histogram gives; on both indexes the confidentiality-only designs and the proposed scheme are indistinguishable. Only the quantum-operator scheme separates, with a histogram variance of 56 619, some 222 times the expected value, and this is the same non-uniformity its chi-square statistic reported in Table 5. The key-space column needs reading with care for a related reason: the nominal space of the MLBPE core is large, yet a one-bit change of its secret parameters leaves the ciphertext unaltered (Table 10), the ISL-HMC scheme retains sensitivity on only three of the six images (Table 11), and the fuzzy scheme derives its context from the plaintext digest with the key text not mixed into it, so the tested key material does not enter the transformation at all; a large nominal key space is therefore not by itself evidence of resistance to key search. Timing is the one genuinely independent axis, and it separates the six schemes by more than an order of magnitude. The two lightest designs are the fuzzy scheme at 51.4 ms per image (10.2 Mbit/s) and the quantum-operator scheme at 62.4 ms (8.4 Mbit/s); the proposed scheme takes 323.7 ms (1.62 Mbit/s); and the three heaviest are the ISL-HMC scheme at 1.623 s, the MLBPE core at 3.590 s and the Hoang structure at 4.009 s, the last of these being twelve times slower than the proposed scheme. The proposed scheme is therefore neither the fastest nor the slowest. It costs about six times the fuzzy scheme, which is what the avalanche and the integrity tag are bought with, and it is an order of magnitude faster than the three chaos-based designs, whose per-pixel keystream generation dominates their running time. The authentication pass accounts for about 0.9 ms per image on the unrounded Table 3 timings, well under one per cent of the proposed scheme’s 323.7 ms, so integrity is very nearly free and the cost sits in the switching and diffusion layers. Robustness to noise and to occlusion, frequently reported as a further index, is deliberately absent for the proposed scheme, and the omission is a property of the design rather than of the evaluation: an authenticated cipher is required to reject any ciphertext that has been altered, so its recovery rate under channel noise or cropping is zero by construction while its rejection rate is one, which is the behaviour reported in Table 13. The five reference schemes will decrypt a corrupted ciphertext into a corrupted image without signalling anything, and that silence is what makes their apparent noise robustness possible; whether it is a feature or a failure depends on whether the receiver is entitled to assume an honest channel. Where partial recovery from a lossy channel is genuinely required, the appropriate construction is an authenticated cipher applied per block, or combined with an error-correcting code, and not a cipher that silently accepts modified input.

### Multi-bit and multi-pixel perturbation

All differential figures reported so far come from flipping one bit at one pixel. That is the minimal perturbation and the one for which the NPCR and UACI thresholds of equations (29) and (30) are derived, but it leaves a question open: whether a scheme that propagates a single bit poorly would propagate a larger change adequately, and conversely whether a scheme that passes on single bits does so only marginally. We therefore repeated the experiment with larger perturbations, one hundred independent trials each: flipping 2, 4 and 8 bits at uniformly chosen positions across the frame, and flipping every bit of 1, 2 and 4 uniformly chosen pixels. The results are collected in Table 16, which reports NPCR; the corresponding UACI and bit-level Hamming distance tables are produced alongside it by the Tables 15–17 program.

**Table 16:** NPCR (%) under perturbations of increasing size, averaged over one hundred independent trials per image and per scheme and then over the six images. The first row is the mean over the six images of the single-bit case of Table 8, computed from the same batch of trials. Under complete avalanche every row should give the same value, 99.6094%.

Perturbation	Proposed	S2 Fuzzy	S3 Quantum	S4 MLBPE	S5 ISL-HMC	S6 Hoang-3
1 bit, 1 pixel	99.6076	99.6012	0.001526	93.3306	99.6102	99.6099
2 bits	99.6093	99.6003	0.003052	96.6473	99.2666	99.6115
4 bits	99.6137	99.6044	0.006104	98.7526	99.6041	99.6109
8 bits	99.6087	99.6055	0.012207	99.5463	99.6080	99.6096
1 pixel (8 bits)	99.5981	99.6040	0.003052	99.6101	99.6085	99.6115
2 pixels	99.5966	99.5979	0.006104	99.6076	99.6097	99.6092
4 pixels	99.5972	99.5984	0.012207	99.6103	99.6090	99.6105

**Table 17:** Distributional diagnostics of the cycle-walked permutation π of the 65 536 pixel positions, compared with the values expected of a uniformly random permutation of the same set. Measured values are means over independent keys.

Diagnostic	Uniform random permutation	Measured
Fixed points	Poisson, mean 1.00	1.34
Cycles	11.67	12.11
Mean |π(i− i|	21 845	21 843.0
Same image row	0.389%	0.3889%
Same 8 × 8 block	0.096%	0.0952%
Destination $\chi^{\text{2}}$ (255 d.f.)	255 ± 22.6	254.0

The interpretation of this table follows from a single observation, which holds independently of the numbers obtained. For a cipher with complete avalanche, the size of the plaintext perturbation is irrelevant: any nonzero difference in the plaintext should make the two ciphertexts differ at each byte position independently with probability $\text{1}\;-\;{\text{2}}^{-\text{8}}$, so every row of Table 16 should give the same 99.6094%, and a row-to-row variation larger than the sampling error is evidence that propagation of a small difference is incomplete. A scheme whose NPCR rises with the number of flipped bits is one in which a single bit reaches only part of the frame and additional flipped bits reach the remainder, which is precisely the failure mode that a single-bit test detects and a multi-bit test conceals.

That is what the table shows, and it separates the two failing schemes in a way the single-bit test alone could not. The quantum-operator scheme does not recover at any perturbation size. Its NPCR is 0.001526% for one flipped bit, 0.003052% for two, 0.006104% for four and 0.012207% for eight, which is 1, 2, 4 and 8 parts in 65 536 to the last digit reported; the pixel rows behave the same way at two bytes per pixel. Each flipped bit changes exactly one output byte and no others, at every perturbation size, so this scheme has no diffusion at all rather than weak diffusion, and no plaintext change short of rewriting the image will produce avalanche in it. The MLBPE core behaves in the opposite way and is the more instructive case. It reaches only 93.3306% on a single bit, then 96.6473%, 98.7526% and 99.5463% on two, four and eight bits, and 99.6101% once every bit of a single pixel is flipped, which is the ideal value. Its diffusion is real but incomplete: a single bit reaches about 93% of the frame, and the remaining 7% is covered as soon as further bits are flipped. A comparison conducted only at whole-pixel perturbations, which is not an unusual choice in this literature, would have recorded this scheme as passing. That is the reason the single-bit case is the one reported in Table 8 and the one the thresholds of equation (29) are derived for.

The remaining schemes are flat, as complete avalanche requires. The proposed scheme lies between 99.5966% and 99.6137% across all seven rows, the fuzzy scheme between 99.5979% and 99.6055%, and the Hoang structure between 99.6092% and 99.6115%; in every case the spread is of the order of the sampling error and carries no information. The ISL-HMC scheme is flat apart from a single dip to 99.2666% at two flipped bits, accompanied by a bit-level Hamming distance of 49.06% rather than 50%. We do not read this as a property of the perturbation size, since the four-bit and eight-bit rows return to 99.6041% and 99.6080%; it is consistent with a small number of trials in which the plaintext-derived context of that scheme collapsed, which is the same instability that leaves it with key sensitivity on only three of the six images in Table 11. The corresponding UACI and bit-level Hamming distance tables, which show the same pattern, are produced by the Tables 15–17 program.

## Discussion

The single most useful outcome of this experiment is negative. Five of the six schemes are indistinguishable on the tests that dominate the image-encryption literature, and the sixth fails them so badly that no careful reader would have needed a comparison to notice. Global entropy, histogram uniformity and adjacent-pixel correlation are saturating measures: once a scheme applies any reasonable byte-level whitening, it will score at the ceiling, and further improvement is measurement noise. Reporting a cipher entropy of 7.9975 rather than 7.9971 as evidence of superiority, as comparison tables routinely do, is reporting the difference between two draws from the same distribution. The same applies to correlation coefficients quoted to five decimal places when the standard error under independence is 0.0039.

Where the schemes separate is on properties that are structural rather than statistical. Plaintext sensitivity separated them because the quantum-operator design has no chaining and no plaintext-dependent key material, so a one-bit change simply cannot propagate; this is visible from the algorithm before any measurement is taken, and the measurement merely confirms it. The MLBPE core separated itself for a subtler reason. Its bit-level avalanche is close to perfect and its byte-level avalanche is not, which produces the unusual signature of a 50.00% bit-change rate coexisting with a 93.33% NPCR. Papers that report only NPCR would call this a failure; papers that report only bit-level diffusion would call it a success. Both would be reporting half of the picture. We include the bit Hamming distance alongside NPCR and UACI precisely so that this kind of disagreement becomes visible.

Key sensitivity is the property on which the comparison is least ambiguous and most damaging. Two of the five reference schemes produced identical ciphertexts under a one-bit key change on every image, and a third did so on half of them. For the fuzzy scheme this reflects a design decision documented in its source: the context is derived from the plaintext digest and the key text is optional. In the configuration described in its source, therefore, the tested key material does not enter the transformation at all, and the secrecy of the ciphertext cannot rest on it. We report this as a property of the configuration we tested and not as a complete cryptanalysis of every configuration of the scheme. For the MLBPE core the failure is a finite-precision artefact. Continuous chaotic systems have positive Lyapunov exponents, so in exact arithmetic a perturbation of $\text{2}\;\times \;{\text{1}\text{0}}^{-\text{1}\text{6}}$ would eventually be amplified; but the amplification is exponential in time with a modest rate, the integration here runs for a fixed 2 000 transient steps at a step size of 0.01, and the output is then quantised by taking the fractional part of a scaled floating-point value modulo 256. Between the finite transient, the fixed step and the quantisation, the perturbation never reaches the output. This mechanism, and not the mathematics of chaos, is what determines key sensitivity in practice, which is why cryptographic key schedules are built from hash functions whose avalanche is a designed property verifiable at the bit level rather than from dynamical systems whose avalanche is an asymptotic one.

The ISL-HMC case is instructive in a different way. Its key sensitivity is not absent but conditional: full avalanche on three images, none on the other three. Because the effective initial condition is the base value plus plaintext-derived quantities reduced modulo one, whether a one-unit-in-the-last-place perturbation of the base survives the reduction depends on the digest of the particular image. A property that holds for half of one’s test set is not a property; had the authors of a scheme with this behaviour tested only the three favourable images, they would have reported ideal key sensitivity in good faith. This is an argument for testing on a fixed public image set rather than one chosen by the proposer.

The integrity results require the least interpretation. Confidentiality without integrity has been understood [22,23] since the 1990s to be an inadequate security goal for transmitted data, and the encrypt-then-MAC composition used here is the standard remedy, with well-known composition theorems behind it. That five recent image-encryption designs still ship without any authentication is not an oversight peculiar to these five papers; it is the norm in this literature, and it is arguably its most serious systematic weakness. An adversary who cannot read a transmitted radiograph but can flip bits in it, with the receiver unable to tell, has an attack that no amount of entropy in the ciphertext prevents. Adding a MAC costs one pass over the data and well under one per cent of the encryption effort, which is why we regard the omission as a design choice that ought to be defended rather than a neutral simplification.

Turning to the proposed scheme, the case for it does not rest on winning the statistical comparison, since on the saturating indicators it merely ties. It rests on the fact that each of its components can be argued for independently. The switching networks are built on a graph with the optimal spectral gap, which gives a rigorous, theorem-backed rate of spatial mixing rather than the heuristic support of a bifurcation diagram, though we stop short of claiming a proof that the complete induced permutation is exactly uniform; the cycle-walking step restricts the permutation from the 68 880 graph vertices to the 65 536 pixel positions without padding and without introducing additional bias relative to the parent permutation, so the ciphertext is exactly as long as the plaintext; the Cauchy matrices are maximum-distance-separable with branch number nine, which is the maximum possible for an 8 × 8 byte transformation and forces a single input byte difference to affect every output byte of its block; the feedback layer is nonlinear because it uses the AES S-box, and it runs in both directions so that dependence is global rather than one-sided; and the whitening and authentication layers are standard primitives used in their standard modes. Nothing in the design requires the reader to accept that a particular map is chaotic on a particular parameter range.

One further point of self-assessment belongs here. With one hundred differential trials per image the proposed scheme passes every cell of Table 14, including the ankle CT slice whose thirty-trial shortfall prompted this part of the revision and which now returns 99.6093%. We are conscious that a comparison in which the proposed method passes everything is the kind of comparison this paper argues against, so we draw attention to the place where the finer-grained data are not in our favour: at trial level the proposed scheme is less stable than two of the reference schemes, with a per-trial standard deviation reaching 0.2785% on Rice and a minimum of 96.8460%, against standard deviations below 0.031% and minima above 99.52% for the ISL-HMC scheme and the Hoang structure. The properties that separate the six designs remain key sensitivity, plaintext propagation and integrity, and on those the separation is structural rather than marginal; but on the stability of its own differential behaviour our scheme is not the best of the six, and the trial-level statistics are reported so that this is visible.

## Limitations

Four limitations should be stated plainly. First, the reference schemes were re-implemented from their published descriptions, and in three cases those descriptions left a step underdetermined: the quantum-operator paper does not fix the image rotation amount or the complete crossover table, the MLBPE paper embeds its core inside a detection-and-inpainting pipeline that is out of scope for a full-frame comparison, and the ISL-HMC paper does not state how the four digest-derived quantities are combined into the two initial values. In each case a fixed, reversible resolution was adopted and is documented in the Methods; a different resolution could change the numbers. The failures we report for the quantum-operator scheme, however, follow from its structure and not from any of the resolved details, since a cipher with no chaining and no plaintext-dependent material cannot have plaintext avalanche whatever crossover table is chosen. We emphasise that this qualification is not confined to the limitations section: it is stated in the Abstract, restated with the experimental protocol, and recorded resolution by resolution in Table 18, because a comparison of re-implementations is a different object from a comparison of published algorithms and the difference should be visible wherever a comparative claim is made.

**Table 18:** Implementation mode and resolution notes for the five reference schemes.

Scheme	Mode	Resolution adopted
S2 Fuzzy [43]	Full published implementation	The published S-box (Table 5 of the source) is used verbatim. The context is derived from the SHA-512 digest of the hexadecimal expansion of the plaintext; the key text is not mixed into the context, which is the configuration described in the source. No nonce.
S3 Quantum [44]	Full published implementation	The source does not fix the image right-rotation amount or the complete 0–F crossover table. A rotation of one nibble and a fixed sixteen-symbol bijective crossover family are used; every resulting nibble lookup table was verified to be bijective.
S4 MLBPE [45]	Encryption core, full frame	The detection and inpainting front end of the source pipeline is outside the scope of a full-frame image comparison and is excluded; the reversible four-stage bit-plane core is applied to the whole grayscale image.
S5 ISL-HMC [46]	Single-image specialisation	The depth parameter is set to one for a single grayscale frame, and the two auxiliary index sequences are generalised accordingly. The source does not state how the four digest-derived quantities are combined into the two initial values; a fixed reproducible pairing is used and the stated base values are retained.
S6 Hoang-3 [47]	Benchmark structural implementation	Permutation, whole-image XOR diffusion and AES S-box substitution, five rounds, with round material derived from SHA-256 of the key text and a round tag.

Second, the test set is six images at a single resolution. Six is enough to expose the failures reported here, and it is more than many published comparisons use, but it is not enough to characterise the tails of any of the statistics. A conclusion of the form “scheme X passes test Y on n of six images” is a statement about a sample of six, not an estimate of a pass rate.

Third, we applied a screening subset of NIST SP 800−22 [48], not the full suite with its recommended sample sizes and second-level uniformity analysis. Seven tests on one 524 288-bit sequence per image detects gross non-randomness and nothing finer. A design intended for deployment should be evaluated with the complete suite over many independent sequences.

Fourth, and most importantly, none of this is cryptanalysis [8,10,12]. Passing a statistical battery is a necessary condition, not a sufficient one, and the history of this field is largely a history of schemes that passed such batteries and were then broken by chosen-plaintext arguments that no statistic would have revealed. The proposed scheme inherits confidentiality from AES-CTR and integrity from HMAC-SHA-256 under standard assumptions, which is a stronger position than a heuristic argument, but the switching, MDS and feedback layers preceding them have not been analysed against differential or linear attacks, and the claim we make for them is a mixing claim rather than a security proof. Independent cryptanalysis remains the necessary next step, and we regard making the complete implementation available as part of that.

## Concluding remarks

Running six image ciphers through one program on one set of pictures produced a result that is easy to state: the tests that everyone reports do not distinguish the schemes, and the tests that distinguish the schemes are largely absent from the literature. Two of five recent designs produced unchanged ciphertexts under a one-bit key change, a third did so on half the images, one had essentially no plaintext sensitivity, and none of the five could tell a modified ciphertext from an intact one. These are not subtle properties requiring elaborate analysis; they are visible in a few hours of controlled measurement. Our recommendation is therefore procedural as much as technical. Comparative claims should be based on re-implementation rather than on numbers copied between papers; key sensitivity should be measured with a one-bit change and reported per image; bit-level and byte-level avalanche should both be reported, because they can disagree; and confidentiality-only designs should be labelled as such. On the constructive side, the components used in the proposed scheme suggest that an image cipher can be assembled from objects with proofs behind them — expander graphs, MDS codes, standard modes and standard MACs — without giving up anything measurable relative to the chaotic designs it is compared against.

## Methods

### Notation and design rationale

Throughout, an image is a two-dimensional array of 8-bit samples of size M × N, read in column-major order into a byte string $x\;=\;(x_{\text{1}},\;\ldots ,\;x_{L})$ of length L = MN. Bytes are elements of the finite field $GF({\text{2}}^{\text{8}})$ whenever they are multiplied and elements of the additive group of the field whenever they are combined by exclusive or, written ⊕. Concatenation of byte strings is written ‖. The symbol S denotes the AES substitution box [20,49] viewed as a permutation of the 256 byte values.

The design principle of the proposed scheme is that every layer should have an independent justification. Position mixing is provided by walks on a graph whose spectral gap is provably optimal; value mixing at the byte level is provided by matrices whose branch number is provably maximal; nonlinearity is provided by an S-box whose differential and linear properties are documented; confidentiality of the final output is provided by a standardised block cipher in a standardised mode; and integrity is provided by a standardised message authentication code. No layer relies on the empirical randomness of a dynamical system.

### Key derivation

The scheme takes a 256-bit master key K and a fresh 96-bit nonce N drawn from a cryptographic random source at every encryption. Four 256-bit subkeys are derived with HKDF in its extract-then-expand form [50,51]. The extraction step is


$$\begin{array}{c} PRK\;=\;{HMAC}_{SHA-\text{2}\text{5}\text{6}}(salt,\;K) \end{array}$$ (1)

where the salt is the fixed 32-byte digest of a domain-separation string, and the expansion step generates the output keying material by the chained recurrence


$$\begin{array}{c} T_{i}\;=\;{HMAC}_{SHA-\text{2}\text{5}\text{6}}\left(PRK,\;T_{i-\text{1}}\;\|\;info\;\|\;i\right),\;i\;=\;\text{1},\;\ldots ,\;\text{4},\;T_{\text{0}}\;=\;\varepsilon \end{array}$$ (2)

with info = "context" ‖ N. The 128-byte output is split into four independent subkeys,


$$\begin{array}{c} OKM\;=\;T_{\text{1}}\;\|\;T_{\text{2}}\;\|\;T_{\text{3}}\;\|\;T_{\text{4}}\;=\;K_{perm}\;\|\;K_{diff}\;\|\;K_{enc}\;\|\;K_{mac} \end{array}$$ (3)

Because the nonce enters the info field, the whole schedule — the three permutations, the two diffusion streams, the counter-mode initial value and the authentication key — is regenerated for every encryption even when the master key and the plaintext are unchanged. All pseudorandom byte streams used below are produced by encrypting zero bytes with AES in counter mode under the relevant subkey and a domain-separated initial value, so that distinct uses of the same subkey never share a keystream.

### The LPS Ramanujan graph

An expander is a sparse graph on which a random walk mixes quickly [14,15]. Quantitatively, if a connected k-regular graph has adjacency eigenvalues $k\;=\;\lambda_{\text{1}}\;\geq \;\lambda_{\text{2}}\;\geq \;\cdots \;\geq \;\lambda_{n}$ and $\lambda \;=\text{ max}(\left|\lambda_{\text{2}}\right|,\;\left|\lambda_{n}\right|)$, the mixing rate is governed by λ, and the Alon–Boppana bound states that λ cannot be smaller than $\text{2}\sqrt{k\;-\;\text{1}}\;-\;o(\text{1})$ as n grows. Graphs attaining $\lambda \;\leq \;\text{2}\sqrt{k\;-\;\text{1}}$ are called Ramanujan, and the Lubotzky–Phillips–Sarnak construction produces an explicit infinite family of them [16,52]. We use the graph $X^{p,q}$ with p = 29 and q = 41, which is (p + 1)-regular, that is 30-regular, and bipartite because p is not a quadratic residue modulo q.

The vertex set is the projective general linear group over the prime field,


$$\begin{array}{c} V\;=\;PGL(\text{2},\;q),\;|V|\;=\;q(q^{\text{2}}\;-\;\text{1}=\;\text{6}\text{8}\;\text{8}\text{8}\text{0} \end{array}$$ (4)

with each class represented canonically by the matrix whose first nonzero entry equals one. The bipartition is by the quadratic character of the determinant, giving two sides of 34 440 vertices each. The generators come from the integral quaternion solutions of the p-th Lagrange equation,


$$\begin{array}{cc} S\;= & \;\{(a_{\text{0}},\;a_{\text{1}},\;a_{\text{2}},\;a_{\text{3}}:\;{a_{\text{0}}}^{\text{2}}\;+\;{a_{\text{1}}}^{\text{2}}\;+\;{a_{\text{2}}}^{\text{2}}\;+\;{a_{\text{3}}}^{\text{2}}\;\\ = & \;p,\;a_{\text{0}}\;>\;\text{0}\;odd,\;a_{\text{1}}\;\equiv \;a_{\text{2}}\;\equiv \;a_{\text{3}}\;\equiv \;\text{0}\;(mod\;\text{2})\},\;|S|\;=\;p\;+\;\text{1}\;=\;\text{3}\text{0} \end{array}$$ (5)

Each solution is mapped to an element of PGL(2, q) through a square root ι of −1 modulo q, which exists because q ≡ 1 (mod 4); for q = 41 one may take ι = 9. The generator matrices are


$$\begin{array}{c} g_{j}\;=\;\left[a_{\text{0}}\;+\;\iota a_{\text{1}}\;a_{\text{2}}\;+\;\iota a_{\text{3}}\;;\;-a_{\text{2}}\;+\;\iota a_{\text{3}}\;a_{\text{0}}\;-\;\iota a_{\text{1}}\right]\;mod\;q,\;j\;=\;\text{1},\;\ldots ,\;\text{3}\text{0} \end{array}$$ (6)

each of which has determinant congruent to p modulo q, and the graph joins every vertex u to the thirty vertices $u\cdot g_{j}$. Because the graph is bipartite and each generator induces a perfect matching between the two sides, each generator defines an involution on the vertex set that always crosses the bipartition. This is the property the switching network exploits.

### Keyed switching networks

A single switch layer is specified by a generator index j and by a vector of independent secret bits b, one bit per vertex of side zero. Writing u for a side-zero vertex and $m_{j}(u)$ for its partner under generator j, the layer acts on a labelling q of the vertices by


$$\begin{array}{c} \sigma_{r}\;:\;(q(u),\;q(m_{j}(u))\mapsto \;\left(q(m_{j}(u)),\;q(u)\right)\;if\;b_{u}\;=\;\text{1},\;and\;identity\;otherwise \end{array}$$ (7)

so that each layer is an involution and therefore trivially invertible. Three independent stages are composed, with 24, 23 and 24 layers respectively, for a total of 71 switch layers:


$$\begin{array}{c} \Pi_{s}\;=\;\sigma_{R_{s}}\;\circ \;\cdots \;\circ \;\sigma_{\text{2}}\;\circ \;\sigma_{\text{1}},\;s\;=\;\text{1},\;\text{2},\;\text{3},\;(R_{\text{1}},\;R_{\text{2}},\;R_{\text{3}}=\;(\text{2}\text{4},\;\text{2}\text{3},\;\text{2}\text{4}) \end{array}$$ (8)

The generator index and the switching bits of every layer are read from the pseudorandom stream derived from $K_{perm}$, so the entire network is a function of the master key and the nonce. The Ramanujan spectral gap provides a rigorous basis for rapid spatial dispersion of labels across the graph, and the optimal gap of the LPS family is what allows a constant number of layers to achieve this. The 71 keyed switching layers provide repeated position mixing; however, the graph spectral bound alone is not taken here as a proof that the complete induced permutation distribution is uniform.

### Cycle walking onto the image domain

The graph has 68 880 vertices, whereas a 256 × 256 image has only L = 65 536 pixels. Each $\Pi_{s}$ therefore permutes a set that is larger than the set of pixel indices, and it cannot be applied to the image as it stands. Padding the image to the vertex count would expand the ciphertext, and truncating the permutation would destroy bijectivity. We use cycle walking instead [53], a technique originally introduced to restrict a cipher on a large domain to an arbitrary smaller one without weakening it. For each index i ≤ L define


$$\begin{array}{c} \pi (i=\;{\Pi_{s}}^{k(i)}(i),\;where\;k(i)\;=\;min\left\{k\;\geq \;\text{1}\;:\;{\Pi_{s}}^{k}(i)\;\leq \;L\right\} \end{array}$$ (9)

The map π is well defined because every point of a finite permutation lies on a cycle that returns to it, and it is a bijection of 1, …, L because the walk is deterministic and reversible. In the implementation the iteration is applied to all out-of-range positions simultaneously and terminates when none remains; the resulting permutation is verified to be bijective before use. Cycle walking preserves the exact length of the plaintext, which is why the ciphertext of a 256 × 256 image is exactly 65 536 bytes.

The present implementation targets 256 × 256 images. For images whose pixel count exceeds the 68 880 vertices of the selected LPS graph, an appropriately larger graph or a defined block-wise extension would be required. Such scaling is outside the scope of the present evaluation.

### Distribution of the cycle-walked permutation

Bijectivity is necessary for correctness but says nothing about mixing, so the distribution of the induced permutation requires a separate argument. That argument has two parts, and it is useful to separate them, because only the first is a theorem. First, cycle walking is distribution-preserving [53]: if Π is distributed uniformly on the permutations of the vertex set V, then the first-return map it induces on any subset D ⊆ V is distributed uniformly on the permutations of D. The restriction therefore introduces no additional bias relative to the parent permutation, and the question of how well π mixes the 65 536 pixel positions reduces entirely to the question of how close the parent permutation Π of the 68 880 graph vertices is to uniform. Second, the parent is produced by a keyed switching network, and for that we have a quantitative bound rather than a proof of uniformity. For a k-regular graph on n vertices with second eigenvalue modulus λ, the distribution of a walk started at any vertex is within total variation distance $\frac{\text{1}}{\text{2}}\sqrt{n}{\left(\lambda /k\right)}^{t}$ of uniform after t steps. For X(29,41) we have n = 68 880, k = 30 and, by the Ramanujan property, $\lambda \;\leq \;\text{2}\sqrt{\text{2}\text{9}}\;\approx \;\text{1}\text{0}.\text{7}\text{7}\text{0}$, so k/λ ≥ 2.786, and for the corresponding simple random walk the bound falls below ${\text{2}}^{-\text{2}\text{0}}$ after approximately 18.3 steps. The implemented switching process is not identical to that walk, so this figure motivates the chosen layer counts of 24, 23 and 24 — each comfortably above it, with the three stages composed — but does not constitute a mixing bound for the switching network itself; its distributional behaviour is therefore assessed empirically in Table 17. What the optimal spectral gap of the LPS graph buys here is a small benchmark for the present graph: approximately 18.3 simple-random-walk steps at the stated total-variation tolerance.

Two limitations of that bound should be stated. It controls the marginal distribution of the destination of a single label, not the joint distribution of the whole permutation. It is also a bound for the simple walk on the graph, whereas each switching layer leaves a label in place whenever its switching bit is unset, so the single-label process is closer to a lazy walk than to the simple one and its mixing is correspondingly slower by a constant factor. We therefore state the bound for what it is: a proved property of the underlying Ramanujan graph, which motivates the number of layers, and not a proof about the implemented switching network. We do not claim that Π is uniform, and the distributional behaviour of the network as implemented is validated empirically below rather than derived. Accordingly the distributional behaviour of π is also measured directly. Table 17 reports six diagnostics, each with the value a uniformly random permutation of 65 536 points would give, computed over independent keys: the number of fixed points, which is Poisson with mean one; the number of cycles, whose expectation is the harmonic number $H_{\text{6}\text{5}\;\text{5}\text{3}\text{6}}\;\approx \;\text{1}\text{1}.\text{6}\text{7}$; the mean displacement |π(i− i|, whose expectation is $\frac{L^{\text{2}}\;-\;\text{1}}{\text{3}L}\;\approx \;\text{2}\text{1}\;\text{8}\text{4}\text{5}$; the fraction of pixels whose image lies in the same image row, taken among non-fixed mappings and expected at $\frac{\text{2}\text{5}\text{5}}{\text{6}\text{5}\;\text{5}\text{3}\text{5}}\;\approx \;\text{0}.\text{3}\text{8}\text{9}\%$; the fraction remaining in the same 8 × 8 block, likewise taken among non-fixed mappings and expected at $\frac{\text{6}\text{3}}{\text{6}\text{5}\;\text{5}\text{3}\text{5}}\;\approx \;\text{0}.\text{0}\text{9}\text{6}\%$; and a destination-bucket chi-square statistic. The last of these needs stating precisely, because a histogram of all 65 536 destinations into 256 buckets is exactly uniform for any permutation and would give zero. Instead the 65 536 source indices are partitioned into 256 equal source buckets; for each source bucket its 256 destinations are histogrammed into 256 equal destination buckets, giving an expected count of one per bucket, and the resulting chi-square statistics on 255 degrees of freedom are averaged over the 256 source buckets. Under a uniform permutation that average has expectation 255.

The cost of the cycle-walking step itself is also easily bounded. Of the 68 880 vertices, 3 344 lie outside the pixel index set, so 4.855% of the vertex space is skipped. Under a uniform parent the number of steps needed for an index to return to the image domain is very closely approximated by a geometric law with success probability L/|V| = 0.9515 — successive positions on a cycle are sampled without replacement, so the law is not exactly geometric — giving a mean walk length close to |V|/L ≈ 1.051 and a probability below ${\text{1}\text{0}}^{-\text{6}}$ of needing more than five steps. The iteration is therefore bounded in practice as well as in principle, and its cost is negligible beside the switching layers themselves.

The measured values come from one hundred reproducible pseudorandom test keys, with the three switching stages averaged within each key, and they sit on their uniform-permutation references. For four of the six diagnostics the measured value is, for practical purposes, the reference value. The mean displacement was 21 843.0 where a uniform permutation gives 21 845.3. Of the pixels that moved, 0.3889% ended up in their original row and 0.0952% in their original 8 × 8 block; the corresponding expectations are 0.3891% and 0.0961%. The destination chi-square came out at 254.0 on 255 degrees of freedom, essentially its expected value. In none of these cases is the measured figure more than about one standard error from the reference. Two diagnostics sit slightly high, and we record them as such rather than round them away: the mean number of fixed points is 1.34 against the Poisson expectation of 1.00, with a standard deviation across keys of 0.80, and the mean number of cycles is 12.11 against 11.67, with a standard deviation of 1.72. The excess in fixed points is small in absolute terms, about one additional pixel in 65 536 left where it started, but it is larger than sampling alone accounts for. It is consistent with what we claim for the construction and not with more: the spectral gap of the underlying graph is proved, but the mixing of the implemented switching network is validated here empirically rather than derived, and a permutation drawn from it is close to but not exactly uniform. The excess is also without cryptographic consequence, because a fixed point of π is not a fixed point of the cipher — the MDS layers, the two-pass feedback diffusion and the counter-mode whitening all act on a byte whether or not the permutation moved it, and the per-stage figures reported by the Tables 15–17 program show the same small excess in each of the three stages rather than in one of them.

### Cauchy MDS diffusion

Permutations move bytes but never change them, so a layer that mixes values is required. We use two applications of a fixed 8 × 8 matrix over the AES field


$$\begin{array}{c} GF({\text{2}}^{\text{8}}=\;F_{\text{2}}[z]\;/\;\left(z^{\text{8}}\;+\;z^{\text{4}}\;+\;z^{\text{3}}\;+\;z\;+\;\text{1}\right) \end{array}$$ (10)

constructed as a Cauchy matrix. Choosing two disjoint sets of field elements $x_{\text{1}},\;\ldots ,\;x_{\text{8}}$ and $y_{\text{1}},\;\ldots ,\;y_{\text{8}}$, the matrix


$$\begin{array}{c} M_{i,j}\;=\;{\left(x_{i}\;⊕\;y_{j}\right)}^{-\text{1}},\;\text{1}\;\leq \;i,\;j\;\leq \;\text{8} \end{array}$$ (11)

has the property that every square submatrix is nonsingular, which makes the associated linear code maximum distance separable [19,21]. Consequently the branch number attains its maximum,


$$\begin{array}{c} B(M=\;{min}_{x\;\neq \;\text{0}}\left\{wt(x)\;+\;wt(Mx)\right\}\;=\;\text{9} \end{array}$$ (12)

so a difference in a single input byte necessarily produces a difference in all eight output bytes of the same block. The byte string is reshaped into blocks of eight and transformed by


$$\begin{array}{c} y\;=\;M\;x,\;y_{i}\;=\;\sum_{j=\text{1}}^{\text{8}}M_{i,j}\;⊗\;x_{j},\;i\;=\;\text{1},\;\ldots ,\;\text{8} \end{array}$$ (13)

where ⊗ is multiplication in $GF({\text{2}}^{\text{8}})$ and the sum is taken with ⊕. The inverse matrix is computed once by Gauss–Jordan elimination over the field and cached; because the field is fixed and small, both M and its inverse are constants of the implementation. In the pipeline an MDS layer is placed between consecutive switching stages, so that positional mixing and value mixing alternate: the permutation carries the eight bytes of one block to eight widely separated positions, and the next MDS layer spreads each of them across a new block.

### Two-pass nonlinear feedback diffusion

The MDS layers are linear, so a nonlinear layer with unbounded dependence range is added. A forward pass computes


$$\begin{array}{c} y_{\text{1}}\;=\;x_{\text{1}}\;⊕\;k_{\text{1}}^{F},\;y_{i}\;=\;x_{i}\;⊕\;k_{i}^{F}\;⊕\;S\left(y_{i-\text{1}}\;⊕\;k_{i-\text{1}}^{F}\right),\;i\;=\;\text{2},\;\ldots ,\;L \end{array}$$ (14)

and a backward pass then computes


$$\begin{array}{c} z_{L}\;=\;y_{L}\;⊕\;k_{L}^{B},\;z_{i}\;=\;y_{i}\;⊕\;k_{i}^{B}\;⊕\;S\left(z_{i+\text{1}}\;⊕\;k_{i+\text{1}}^{B}\right),\;i\;=\;L-\text{1},\;\ldots ,\;\text{1} \end{array}$$ (15)

where $k^{F}$ and $k^{B}$ are independent keystreams of length L derived from $K_{diff}$ under distinct domain separators. Each recurrence is invertible because the feedback term at position i depends only on values that are already known when that position is processed in the reverse direction; explicitly, the forward pass is undone by


$$\begin{array}{c} x_{i}\;=\;y_{i}\;⊕\;k_{i}^{F}\;⊕\;S\left(y_{i-\text{1}}\;⊕\;k_{i-\text{1}}^{F}\right),\;i\;=\;\text{2},\;\ldots ,\;L \end{array}$$ (16)

and symmetrically for the backward pass. Running the recursion in both directions makes every output byte depend on every input byte: the forward pass propagates dependence from low to high indices and the backward pass from high to low, so the composition has full dependence in both directions after a single application, which a one-directional chain cannot achieve.

### Whitening, authentication and the complete cipher

The transformed byte string is finally encrypted with AES in counter mode [49,54]. The counter-mode initial value is derived from the nonce and a domain string,


$$\begin{array}{c} IV\;=\;{trunc}_{\text{1}\text{6}}\left(SHA-\text{2}\text{5}\text{6}\left(N\;\|\;D\right)\right),\;C\;=\;z\;⊕\;{AES}_{K}^{CTR}(IV) \end{array}$$ (17)

and the ciphertext is authenticated in the encrypt-then-MAC arrangement [22,23],


$$\begin{array}{c} \tau \;=\;{HMAC}_{SHA-\text{2}\text{5}\text{6}}\left(K_{mac},\;hdr\;\|\;N\;\|\;C\right) \end{array}$$ (18)

where hdr is a fixed version string together with the image dimensions in big-endian form. The transmitted package is the triple (N, C, τ). Encrypt-then-MAC is chosen because it is the composition that yields authenticated encryption from a secure encryption scheme and a secure MAC without additional assumptions. The complete encryption map is therefore


$$\begin{array}{c} C\;=\;W\;\circ \;F\;\circ \;\Pi_{\text{3}}\;\circ \;M\;\circ \;\Pi_{\text{2}}\;\circ \;M\;\circ \;\Pi_{\text{1}}(x),\;E(x=\;(N,\;C,\;\tau ) \end{array}$$ (19)

with M the MDS layer, F the two-pass feedback diffusion and W the counter-mode whitening. Decryption verifies the tag first, in constant time, and returns a failure symbol without performing any inverse transformation if the comparison fails; otherwise it applies


$$\begin{array}{c} x\;=\;{\Pi_{\text{1}}}^{-\text{1}}\;\circ \;M^{-\text{1}}\;\circ \;{\Pi_{\text{2}}}^{-\text{1}}\;\circ \;M^{-\text{1}}\;\circ \;{\Pi_{\text{3}}}^{-\text{1}}\;\circ \;F^{-\text{1}}\;\circ \;W^{-\text{1}}(C) \end{array}$$ (20)

Since every layer is a bijection of the set of byte strings of length L, decryption is exact and lossless; this was verified for all six images and all thirty-six image–scheme combinations before any statistic was computed. The key space is


$$\begin{array}{c} |K|\;=\;{\text{2}}^{\text{2}\text{5}\text{6}}\;\approx \;\text{1}.\text{1}\text{6}\;\times \;{\text{1}\text{0}}^{\text{7}\text{7}} \end{array}$$ (21)

which is far beyond exhaustive search, and the 96-bit nonce space makes accidental nonce reuse negligible for any realistic volume of traffic.

### Algorithmic description

For completeness, and so that the construction can be re-implemented without reference to the prose, the encryption and decryption procedures are stated as explicit algorithms. Algorithm 1 is the complete encryption map of equation (19) together with the key schedule and the tag; Algorithm 2 is its inverse, equation (20), preceded by the tag verification that gates it. The notation is that of the preceding subsections, except that the 96-bit nonce is written $N_{c}$ in the algorithms so that N is reserved throughout for the image dimension, and the equation numbers in the right-hand comments point to the definition of each step.

**Algorithm 1. RAPID-Gray encryption.**

Input: grayscale image P of size M × N, L = MN; 256-bit master key K.

Output: transmitted package (N_c_, C, τ).

1.  N_c_ ← 96 fresh random bits drawn from a cryptographic source.

2.  PRK ← HMAC-SHA-256(salt, K). ▷ eq. (1)

3.  (K_perm_, K_diff_, K_enc_, K_mac_) ← HKDF-Expand(PRK, ‘context’ ‖ N_c_, 128 bytes). ▷ eqs. (2)–(3)

4.  x ← column-major byte string of P, so x ∈ {0, …, 255}^L^.

5. Build or load the LPS graph X(29,41); verify |V| = 68 880, a balanced bipartition, det(g_j_) ≡ p (mod q) for all j, and that every generator induces a perfect matching across the bipartition. ▷ eqs. (4)–(6)

6.  for s = 1, 2, 3 do

7.  Read the generator index and the switching bits of each of R_s_ layers from the AES-CTR stream under K_perm_, with (R_1_, R_2_, R_3_) = (24, 23, 24).

8.  Π_s_ ← σ_Rs_ ∘ ⋯ ∘ σ_1_. ▷ eqs. (7)–(8)

9.  π_s_ ← cycle-walk(Π_s_) restricted to {1, …, L}; verify bijectivity. ▷ eq. (9)

10. end for

11. x ← π_1_(x); x ← M·x; x ← π_2_(x); x ← M·x; x ← π_3_(x). ▷ eqs. (11)–(13)

12. Derive keystreams k_F_ and k_B_ of length L from K_diff_ under distinct domain separators.

13. y ← forward-pass(x, k_F_); z ← backward-pass(y, k_B_). ▷ eqs. (14)–(15)

14. IV ← trunc_16_(SHA-256(N_c_ ‖ D)); C ← z ⊕ AES-CTR_Kenc_(IV). ▷ eq. (17)

15. τ ← HMAC-SHA-256(K_mac_, hdr ‖ N_c_ ‖ C). ▷ eq. (18)

16. return (N_c_, C, τ).

**Algorithm 2. RAPID-Gray decryption with tag verification.**

Input: package (N_c_, C, τ); 256-bit master key K.

Output: the image P, or the failure symbol ⊥.

1.  Re-derive (K_perm_, K_diff_, K_enc_, K_mac_) from K and N_c_ exactly as in steps 2–3 of Algorithm 1.

2.  τ′ ← HMAC-SHA-256(K_mac_, hdr ‖ N_c_ ‖ C).

3.  if τ′ ≠ τ under a constant-time comparison then return ⊥. ▷ no inverse layer is executed

4.  IV ← trunc_16_(SHA-256(N_c_ ‖ D)); z ← C ⊕ AES-CTR_Kenc_(IV).

5.  Derive k_F_ and k_B_ from K_diff_ as in step 12 of Algorithm 1.

6.  y ← backward-pass^−1^(z, k_B_); x ← forward-pass^−1^(y, k_F_). ▷ eq. (16)

7.  Rebuild π_1_, π_2_, π_3_ from K_perm_ exactly as in steps 5–10 of Algorithm 1.

8.  x ← π_3_^−1^(x); x ← M^−1^·x; x ← π_2_^−1^(x); x ← M^−1^·x; x ← π_1_^−1^(x).

9.  return P ← reshape(x, M × N).

Every step of Algorithm 1 is a bijection on byte strings of length L, so Algorithm 2 recovers P exactly whenever the tag verifies, and returns ⊥ otherwise, without performing any inverse transformation or releasing any plaintext. The work per image is 71 switching involutions, two MDS passes, two feedback passes, one AES-CTR pass and one HMAC pass over L bytes, giving Θ(L) work with small constants; the key schedule is independent of the image and its cost is amortised over the frame.

### Reference implementations and fidelity notes

The five reference schemes were implemented from their published descriptions [43–47] and were not modified by the comparison harness; the harness only calls their encryption, decryption and context-preparation routines and applies the common test battery to their outputs. Where a description was genuinely ambiguous, a fixed reversible resolution was chosen and is recorded here so that the reported numbers can be attributed correctly.

Two of the reference schemes derive their context from the plaintext. For those two, the differential trials regenerate the context from the modified plaintext, because that is what the algorithms specify; holding it fixed would have measured a different function from the one published. For the proposed scheme the nonce and context are held fixed across a differential trial, which isolates plaintext sensitivity from the randomisation that the nonce provides; nonce sensitivity is measured separately.

### Evaluation metrics

All statistics were computed by a single implementation applied identically to every scheme. Global Shannon entropy [55] of an 8-bit image is


$$\begin{array}{c} H(X=\;-\;\sum_{i=\text{0}}^{\text{2}\text{5}\text{5}}p_{i}\;{log}_{\text{2}}\;p_{i},\;p_{i}\;=\;\frac{n_{i}}{MN} \end{array}$$ (22)

where $n_{i}$ is the number of pixels of intensity i. Local entropy [56] is the same quantity evaluated on non-overlapping blocks and averaged,


$$\begin{array}{c} H_{loc}\;=\;\frac{\text{1}}{B}\sum_{b=\text{1}}^{B}H\left(X_{b}\right),\;X_{b}\;a\;\text{1}\text{6}\;\times \;\text{1}\text{6}\;block \end{array}$$ (23)

Histogram uniformity is assessed with the chi-square statistic


$$\begin{array}{c} \chi^{\text{2}}\;=\;\sum_{i=\text{0}}^{\text{2}\text{5}\text{5}}\frac{{\left(n_{i}\;-\;e\right)}^{\text{2}}}{e},\;e\;=\;\frac{MN}{\text{2}\text{5}\text{6}} \end{array}$$ (24)

which has 255 degrees of freedom under the hypothesis of a uniform histogram. Adjacent-pixel correlation for a set of T sampled pairs $\left(u_{t},\;v_{t}\right)$ with sample means $\mu_{u}$ and $\mu_{v}$ is the Pearson coefficient


$$\begin{array}{c} \;=\;\frac{\sum_{t=\text{1}}^{T}\left(u_{t}\;-\;\mu_{u}\mathrm{\backslash rightleft}(v_{t}\;-\;\mu_{v}\right)}{\sqrt{\sum_{t=\text{1}}^{T}{\left(u_{t}\;-\;\mu_{u}\right)}^{\text{2}}\;\sum_{t=\text{1}}^{T}{\left(v_{t}\;-\;\mu_{v}\right)}^{\text{2}}}} \end{array}$$ (25)

evaluated over all horizontally, vertically and diagonally adjacent pairs of the image. Differential behaviour is quantified by the number of pixels change rate and the unified average changing intensity [57] between two ciphertexts $C^{\text{1}}$ and $C^{\text{2}}$ obtained from plaintexts differing in one bit,


$$\begin{array}{c} NPCR\;=\;\frac{\text{1}\text{0}\text{0}}{MN}\sum_{i,j}D(i,\;j),\;D(i,\;j=\;\text{1}\;if\;C^{\text{1}}(i,\;j)\;\neq \;C^{\text{2}}(i,\;j)\;and\;\text{0}\;otherwise \end{array}$$ (26)

$$\begin{array}{c} UACI\;=\;\frac{\text{1}\text{0}\text{0}}{MN}\sum_{i,j}\frac{\left|C^{\text{1}}(i,\;j-\;C^{\text{2}}(i,\;j)\right|}{\text{2}\text{5}\text{5}} \end{array}$$ (27)

and by the bit-level Hamming distance, which we report alongside them because the two can disagree,


$$\begin{array}{c} BHD\;=\;\frac{\text{1}\text{0}\text{0}}{\text{8}MN}\;wt\left(C^{\text{1}}\;⊕\;C^{\text{2}}\right) \end{array}$$ (28)

with wt the Hamming weight of a byte string. For a 256 × 256 8-bit image the one-sided NPCR critical value and the two-sided UACI acceptance interval at level α are


$$\begin{array}{c} N_{\alpha }^{*}\;=\;\frac{Q\;-\;\Phi^{-\text{1}}(\alpha \sqrt{Q\;/\;MN}}{Q\;+\;\text{1}},\;Q\;=\;\text{2}\text{5}\text{5} \end{array}$$ (29)

$$\begin{array}{c} U_{\alpha }^{*}\;=\;\frac{Q\;+\;\text{2}}{\text{3}Q\;+\;\text{3}}\;\mp \;\Phi^{-\text{1}}(\alpha \;/\;\text{2}\sqrt{\frac{\left(Q\;+\;\text{2})(Q^{\text{2}}\;+\;\text{2}Q\;+\;\text{3}\right)}{\text{1}\text{8}(Q\;+\;\text{1})Q^{\text{2}}\;MN}} \end{array}$$ (30)

giving 99.5693% and [33.2824, 33.6447]% at α = 0.05. Plaintext-to-ciphertext distortion is reported as


$$\begin{array}{c} PSNR\;=\;\text{1}\text{0}\;{log}_{\text{1}\text{0}}\frac{{\text{2}\text{5}\text{5}}^{\text{2}}}{MSE},\;MSE\;=\;\frac{\text{1}}{MN}\sum_{i,j}{\left(P(i,\;j-\;C(i,\;j)\right)}^{\text{2}} \end{array}$$ (31)

together with the structural similarity index of Wang and co-workers [58], computed with its standard parameters. Randomness screening uses the frequency, block frequency, runs, spectral, approximate entropy and serial statistics of NIST SP 800−22 [48] applied to the bit expansion of each cipher image; for example the frequency test statistic and p-value are


$$\begin{array}{c} s_{obs}\;=\;\frac{\left|\sum_{i=\text{1}}^{n}\varepsilon_{i}\right|}{\sqrt{n}},\;p\;=\;erfc\left(\frac{s_{obs}}{\sqrt{\text{2}}}\right),\;\varepsilon_{i}\;\in \;\left\{-\text{1},\;+\text{1}\right\} \end{array}$$ (32)

with the remaining statistics as defined in the specification [48]. A p-value below 0.01 is treated as a failure of that test for that sequence.

The additional indexes of Table 15 are defined as follows. The mean squared error between the plaintext and the ciphertext is the quantity MSE already used in equation (31). The variance of the cipher histogram is $\text{var}(h=\;\frac{\text{1}}{\text{2}\text{5}\text{6}}\sum_{i}{\left(n_{i}\;-\;e\right)}^{\text{2}}$ with $e\;=\;\frac{MN}{\text{2}\text{5}\text{6}}$, which is the chi-square statistic of equation (24) multiplied by e/256 and is reported because it is the form in which this index usually appears. Encryption time is wall-clock time for one 256 × 256 image, measured as the mean of ten consecutive encryptions after one discarded warm-up call, with key derivation included and file input and output excluded; throughput is 8MN divided by that time. All timings were taken on the single machine described in the reproducibility subsection, with every scheme measured in the same session, so they are comparable with one another even though absolute values are implementation-dependent and should not be compared with times published elsewhere.

Equations (29) and (30) give thresholds for a single pair of ciphertexts, and the differential figures reported in this paper are means over n independent trials, so the corresponding criterion must be restated for the mean. Under the ideal model in which the two ciphertexts differ independently at each byte position with probability $\text{1}\;-\;{\text{2}}^{-\text{8}}$, a single-trial NPCR is a scaled binomial with mean 99.6094% and standard deviation $\sqrt{p(\text{1}\;-\;p)/MN}\;=\;\text{0}.\text{0}\text{2}\text{4}\text{4}\%$ for a 256 × 256 image; the mean of n such trials has standard error $\text{0}.\text{0}\text{2}\text{4}\text{4}\%/\sqrt{n}$, which is 0.0024% at n = 100. The appropriate one-sided 5% bound for the trial mean is therefore $\text{9}\text{9}.\text{6}\text{0}\text{9}\text{4}\;-\;\text{1}.\text{6}\text{4}\text{5}\;\times \;\text{0}.\text{0}\text{2}\text{4}\text{4}/\sqrt{n}$ per cent, which is 99.6054% at n = 100 and is far tighter than the single-trial value 99.5693%. We report both, together with the z-statistic $(\overset{―}{x}\;-\;\text{9}\text{9}.\text{6}\text{0}\text{9}\text{4}sqrtn/\text{0}.\text{0}\text{2}\text{4}\text{4}$, and we also report the per-image minimum over trials and the count of individual trials exceeding the single-trial threshold, because a mean depressed by a few outlying trials and a mean depressed uniformly are different phenomena that a single averaged number cannot distinguish. The same restatement applies to UACI, whose single-trial standard deviation under the ideal model is obtained from equation (30) in the same way. One consequence must be stated explicitly, because the two criteria would otherwise conflict. Table 14 records the conventional decision, the tabulated mean against the single-trial critical value, and we retain it for one reason: it is the rule under which every scheme in this comparison was judged in its own paper, and substituting a different rule would make the comparison incommensurable with the literature it is meant to test. We state plainly that it is not a valid significance test for an averaged quantity. We therefore report, for every image and scheme, the standard deviation across trials, the smallest NPCR observed in any trial, and the number of the hundred trials that individually exceed the single-trial critical value, which an ideal cipher passes about ninety-five times in a hundred. That count is a proper test at the level at which the critical value is defined, it is reported alongside every mean, and where it disagrees with the mean-based decision we say so in the text. The tightened bound for the trial mean and the z-statistic are reported as descriptive measures of distance from the ideal. The same convention applies to UACI.

The extended differential protocol used for Table 16 is defined as follows. For a perturbation of b bits, b distinct bit positions are drawn uniformly without replacement from the 8MN bit positions of the plaintext and flipped; for a perturbation of p pixels, p distinct pixel positions are drawn uniformly without replacement and all eight bits of each are flipped, which is the largest change that can be made at a fixed number of pixels. In every case the secret material and, for the proposed scheme, the nonce are held fixed across the pair, and for the two schemes whose context is derived from the plaintext the context is regenerated from the modified plaintext, exactly as in the single-bit protocol. One hundred independent trials are run for each perturbation size, each image and each scheme, and the statistics of equations (26) to (28) are computed on each pair. The ideal values are the same as in the single-bit case, because they do not depend on the size of the plaintext difference.

### Implementation and reproducibility

All experiments reported in this paper were run in MATLAB R2020a on a single machine: an Intel Core i5-6200U processor at 2.30 GHz, with an observed base clock of 2.40 GHz, 8 GB of installed memory of which 7.88 GB is usable, under 64-bit Windows 10 Home. Every scheme was measured on that machine, in the same session, with no other user process running, which is what makes the timings in Tables 3 and 15 comparable with one another. They are not comparable with times published elsewhere, and they should not be read as a ceiling on what any of the six designs can achieve: the machine is a dual-core mobile processor of 2015 vintage, the implementations are single-threaded MATLAB rather than optimised native code, and the proposed scheme in particular calls the Java security provider for AES and HMAC through a byte-conversion layer. What the timings establish is the relative cost of the six designs under identical conditions, which is the only timing claim this paper makes.

Everything reported here was produced by MATLAB programs that contain the proposed scheme, the five reference schemes and the common test battery. The cryptographic primitives — AES in counter mode, SHA-256, SHA-512 and HMAC-SHA-256 — are taken from the Java security provider available inside MATLAB, with a bit-preserving byte conversion that was verified by a round-trip self-test before any measurement; the naive conversion from unsigned to signed bytes saturates values above 127 in MATLAB and would silently corrupt every cryptographic operation, so this test is not a formality. A second self-test verifies the invertibility of each permutation stage, of the MDS layer, of the two-pass diffusion, of the counter-mode round trip, of the complete pre-authentication transform and of the complete authenticated package before the image loop begins.

The graph is constructed once and cached, and its construction is verified at run time: the enumeration of PGL(2,41) is checked against the group order, the bipartition is checked to be balanced, every generator is checked to have determinant congruent to p modulo q, and every generator is checked to induce a perfect matching that crosses the bipartition. Each cycle-walked permutation is checked to be bijective. The differential trials use one hundred independent uniformly chosen bit positions per image and per scheme, and the extended perturbation trials of Table 16 use the same number at each perturbation size. Three MATLAB programs were used, and their provenance is stated here so that every table can be traced: a six-scheme comparison program, which produces Tables 2 and 4 to 14 and Figs 2-6; an ablation program, which produces Table 3; and an additional-experiments program, which produces Tables 8, 9, 15–17. Together they regenerate every number in this paper, and all three are supplied as S1 Code; because the nonce of the proposed scheme and the bit positions of the differential trials are drawn fresh on each run, trial-dependent statistics reproduce in distribution rather than digit-for-digit, while every deterministic statistic reproduces exactly.

## Supporting information

S1 Code RAPID gray all experiments. (ZIP)
